# Double-Stranded RNA Viruses Are Released From *Trichomonas vaginalis* Inside Small Extracellular Vesicles and Modulate the Exosomal Cargo

**DOI:** 10.3389/fmicb.2022.893692

**Published:** 2022-05-04

**Authors:** Petr Rada, Ivan Hrdý, Alois Zdrha, Ravi Kumar Narayanasamy, Tamara Smutná, Jana Horáčková, Karel Harant, Vladimír Beneš, Seow-Chin Ong, Chih-Yu Tsai, Hong-Wei Luo, Cheng-Hsun Chiu, Petrus Tang, Jan Tachezy

**Affiliations:** ^1^Department of Parasitology, Faculty of Science, Charles University, Biotechnology and Biomedicine Center in Vestec (BIOCEV), Vestec, Czechia; ^2^Genomics Core Facility, European Molecular Biology Laboratory, Heidelberg, Germany; ^3^Department of Parasitology, College of Medicine, Chang Gung University, Taoyuan, Taiwan; ^4^Molecular Infectious Disease Research Center, Chang Gung Memorial Hospital, Linkou, Taiwan

**Keywords:** *Trichomonasvirus*, TVV, exosome, extracellular vesicle, proteomics, tsRNA

## Abstract

*Trichomonas vaginalis* is a parasitic protist that infects the human urogenital tract. During the infection, trichomonads adhere to the host mucosa, acquire nutrients from the vaginal/prostate environment, and release small extracellular vesicles (sEVs) that contribute to the trichomonad adherence and modulate the host-parasite communication. Approximately 40–70% of *T. vaginalis* strains harbor a double-stranded RNA virus called *Trichomonasvirus* (TVV). Naked TVV particles have the potential to stimulate a proinflammatory response in human cells, however, the mode of TVV release from trichomonads to the environment is not clear. In this report, we showed for the first time that TVV particles are released from *T. vaginalis* cells within sEVs. The sEVs loaded with TVV stimulated a higher proinflammatory response of human HaCaT cells in comparison to sEVs from TVV negative parasites. Moreover, a comparison of *T. vaginalis* isogenic TVV plus and TVV minus clones revealed a significant impact of TVV infection on the sEV proteome and RNA cargo. Small EVs from TVV positive trichomonads contained 12 enriched and 8 unique proteins including membrane-associated BspA adhesine, and about a 2.5-fold increase in the content of small regulatory tsRNA. As *T. vaginalis* isolates are frequently infected with TVV, the release of TVV *via* sEVs to the environment represents an important factor with the potential to enhance inflammation-related pathogenesis during trichomoniasis.

## Introduction

*Trichomonas vaginalis* is a causative agent of human trichomoniasis, a sexually transmitted disease with an incidence of about 156 million cases worldwide (World Health Organization [WHO], 2018). Symptomatic infection is observed in approximately 50% of women that can experience vaginitis and urethritis (Sutton et al., 2007). In addition, trichomoniasis is associated with adverse outcomes during pregnancy and may contribute to pelvic inflammatory disease (Silver et al., 2014; Yagur et al., 2021). In men, *T. vaginalis* infection is in over 75% of cases asymptomatic with parasites hidden in the prostate that represent a reservoir for transmission (Hirt and Sherrard, 2015). The symptomatic infections include urethritis and prostatitis, and the trichomonads have been found in prostate tissue from benign prostatic hyperplasia (Mitteregger et al., 2012; Van Gerwen et al., 2021). Moreover, trichomoniasis is associated with an increased risk of cervical and prostate cancer and transmission of HPV and HIV, which is related to erosion of the mechanical barrier and modulation of immune status (Kissinger and Adamski, 2013; Twu et al., 2014; Belfort et al., 2021; Kushwaha et al., 2021; Van Gerwen et al., 2021).

Multiple virulent factors participate in the parasite interactions with the host cells and vaginal microbiota to orchestrate the parasite establishment and pathogenesis. Most biologically active molecules are secreted *via* the classical endoplasmic reticulum-Golgi complex secretory pathway (Stáfková et al., 2018). However, trichomonads employ at least two unconventional protein secretion (UPS) pathways *via* lysosomes and extracellular vesicles (EVs; Twu et al., 2013; Rabouille, 2017; Zimmann et al., 2021). Particularly, EVs have been described to play a critical role in cell-cell communication including pathophysiological situations caused by parasitic protists (Dong et al., 2019; Dias-Guerreiro et al., 2021; Opadokun and Rohrbach, 2021). Two types of EVs were characterized in *T. vaginalis*: small vesicles of 50–150 nm in diameter (exosomes) that are formed intracellularly in the lumen of endosomal multivesicular bodies (Twu et al., 2013), and larger vesicles over 200 nm that bud directly from the trichomonad plasma membrane (Nievas et al., 2018). Both EV types were shown to mediate parasite-parasite and parasite-human epithelial cells interactions (Twu et al., 2013; Nievas et al., 2018). *T. vaginalis* exosomes contain over 200 proteins and small size RNAs, particularly tsRNA (Twu et al., 2013; Artuyants et al., 2020). The vesicles interact with glycosaminoglycans at the membrane of the target cells and internalize *via* lipid raft-mediated endocytosis to deliver their cargo (Rai and Johnson, 2019). Interactions of isolated EVs with human cells *in vitro*, as well as intravaginal inoculation of EVs in a murine model, revealed their immunomodulatory properties (Twu et al., 2013; Olmos-Ortiz et al., 2017). Moreover, EVs are directly involved in the adherence of trichomonads to the epithelial cells, an important process for the establishment of *T. vaginalis* infection and pathologies (Twu et al., 2013).

In addition to endogenous virulence factors, *T. vaginalis* cells frequently contain a dsRNA virus named *Trichomonasvirus* (TVV) of Totiviridae group that was suspected to modulate the outcome of *T. vaginalis* infection. The presence of TVV may alter gene expression in *T. vaginalis* as documented by the increased level of surface immunogenic protein P270, and changes in cysteine proteinase expression profiles in the infected parasite (Alderete, 1999). However, the clinical relevance of these observations remains unclear. There are four TVV species (TVV1-4) of which TVV1 and TVV2 have been associated with more severe clinical symptoms (Fraga et al., 2012), however, a significant relationship between the symptoms and the presence of TVVs has not been found in other studies (Graves et al., 2019). TVVs harbor non-segmented genomes of 4.3–5.5 kb, which are enclosed in a protective, single-layered icosahedral capsid of 32–45 nm in diameter (Flegr et al., 1987; Parent et al., 2013; Stevens et al., 2021). The genomes encode a capsid protein (CP) of 72–90 kDa (Liu et al., 1998; Bessarab et al., 2011), and RNA-dependent RNA polymerase (RdRp) that is expressed as a fusion protein of 160 kDa with N-terminal CP (Liu et al., 1998). Like other members of the Totiviridae group, TVVs are transmitted vertically to the daughter cells during cell division (Kuhlmann et al., 2017; Narayanasamy et al., 2021), while extracellular transmission *via* lysis of the host cells seems to be absent. Although *T. vaginalis* may engulf isolated viral particles (Benchimol et al., 2002), this process did not lead to stable trichomonad infection (Wang and Wang, 1986).

The exosomes shed by TVV-infected parasites may represent a potential novel tool for TVV communication with the microenvironment as observed for other viruses (Ju et al., 2021). For example, the dsRNA virus in *Leishmania guyanensis* (LRV1) has been implicated to be a key virulence factor for the development of mucocutaneous leishmaniasis (Ives et al., 2011; Atayde et al., 2019). LTV1 has a major impact on the protein content of parasite’s exosomes *via* modulation of mRNA translation and exploits *Leishmania* for packing viral particles into the exosomes to be released into the extracellular environment (Atayde et al., 2019). LRV1, a member of Totiviridae (Goodman et al., 2011), is a TVV relative, thus a packaging of TVV particles in exosomes could be expected. However, a recent proteomic analysis of EVs derived from TVV-positive *T. vaginalis* strains did not identify proteins of TVV origin in Evs (Govender et al., 2020). Interestingly, EVs from TVV-positive strains were immunosuppressive to human epithelial cells, whereas proinflammatory response was observed upon the cell interaction with TVV genomic dsRNA, and a synthetic dsRNA analog (Fichorova et al., 2012; Govender et al., 2020).

Recently, our group derived from *T. vaginalis* TVV infected strain isogenic clones with and without TVV endobionts using 2′-C-methylcytidine (2′CMC), an inhibitor of RdRp (Narayanasamy et al., 2021). Herein, we exploit these clones for comparative investigations of an effect of the endosymbiotic virus on the cargo of small exosomal vesicles (sEVs) including proteomic and RNA deep sequencing analysis. We will use the term sEVs instead of exosomes based on isolation methods used in this study as recommended (Kowal et al., 2016). Our results provide evidence that TVV particles, proteins, and RNA of viral origin are exported within sEVs to the extracellular environment. We also demonstrated significant changes in protein and RNA cargo between EVs from TVV positive and TVV negative clones. Moreover, sEVs derived from TVV-positive clones caused higher pro-inflammatory responses in epithelial cells than those from TVV-negative trichomonads. Our results suggest that TVV virions and associated changes in sEV cargo may represent an increased risk for inflammation-dependent symptoms of trichomononiasis and pathogenic inflammatory responses linked to other associated morbidities.

## Materials and Methods

### Cells and Cultivation

The *T. vaginalis* clones containing TVV1, TVV2, and TVV3 viruses (TV79-49c1^+^) and isogenic TVV-free clone TV79-49c1^–^ were used in this study (Narayanasamy et al., 2021). The TVV-free clone was derived from TV79-49c1^+^ by 2′-C-methylcytidine treatment as described (Narayanasamy et al., 2021). Trichomonads were cultivated in the tryptone-yeast extract-maltose (TYM) medium at pH 6.2, supplemented with 10% inactivated horse serum (Gibco Life Technologies, Penrose, New Zealand) at 37°C (Diamond, 1957). Human spontaneously transformed aneuploid immortal keratinocyte cells (HaCaT, a kind gift of V. Vonka, Institute of Hematology and Blood Transfusion, Prague; RRID:CVCL_0038; Vonka et al., 1995) were grown in RPMI 1640 complete medium supplemented with 10% fetal bovine serum, 2‰ NaHCO_3_, penicillin (100 U/ml), and streptomycin (100 μl/ml). The absence of mycoplasma contamination was tested by PCR as described (Van Kuppeveld et al., 1994).

### Preparation of Polyclonal Antibody Against Tetraspanin 1

A partial gene coding for 78 amino acid residues of the soluble domain of transmembrane protein Tsp1 (TVAG_019180) was amplified from *T. vaginalis* genomic DNA by PCR (primers are given in Supplementary Table 1) and cloned into bacterial vector pET42b (Novagen, Sigma-Aldrich, St. Louis, Unites States). Recombinant Tsp1 peptide was expressed with His-tag in *Escherichia coli* strain DE3 using autoinduction medium (Studier, 2005) and affinity purified with His-tagged fusion proteins purification system (ThermoFisher Scientific, Waltham, Unites States). Purified protein was used for immunization of Wistar Han IGS rats (BIOCEV Core facility, Vestec, Czechia), and polyclonal anti Tsp1 antibody was registered in Antibody Registry (RRID:AB_2910119).

### Isolation of Small Exosomal Vesicles

The *T. vaginalis* culture of 1 × 10^9^ cells in the logarithmic phase of growth was harvested by centrifugation for 15 min at 2000 × *g* and 25°C, and the cell pellet was washed twice with serum-free TYM medium. Washed trichomonads were resuspended in serum-free TYM medium to a concentration of 1 × 10^6^ cells/ml and incubated for 2.5 h at 37°C. Cell integrity was checked microscopically using the trypan blue exclusion test (Strober, 2015). Then, the cells were pelleted by centrifugation for 15 min at 2000 × *g* and 25°C. The supernatant with exosomes and other vesicles was filtered through a 0.22 μm pore size filter to remove any non-sedimented cells. The filtered supernatant was concentrated by tangential filtration using a Vivaflow 200 system with 100,000 MWCO PES membrane (Sartorius, Göttingen, Germany) to a final volume of approximately 30 ml. The concentrated filtrate was centrifuged for 1 h at 100,000 × *g* and 4°C, and proteins in the supernatant were precipitated with methanol and chloroform (4:1). The precipitate was analyzed by mass spectrometry to subtract contaminating proteins from EV proteome. The pellet (EV) containing sEVs was resuspended in Sucrose-Tris (ST) buffer (250 mM sucrose, 10 mM Tris, 0.5 mM KCl, pH 7.2) and fractionated on a linear gradient of 5–50% OptiPrep (5% OptiPrep was made using 250 mM sucrose, 1 mM EDTA, 10 mM Tris, pH 7.4, and 50% OptiPrep was prepared using 250 mM sucrose, 6 mM EDTA, 60 mM Tris, pH 7.4) according to OptiPrep™protocol (Axis-Shield PoC AS, Norway) on a Beckman SW 41 swing-out rotor at 100,000 × *g* and 4°C for 15 h. The gradient was fractionated using a Beckman Fraction recovery system (Beckman Coulter Inc., Brea, United States) connected to an FPLC pump, a UV-Vis flow spectrophotometer, and a fraction collector. The individual gradient fractions (0.5 ml) were diluted at 1:10 in ST buffer and centrifuged for 1 h at 100,000 × *g* and 4°C. The washed pellets were analyzed by immunoblotting using antibodies against TVV1 (RRID:AB_2910120) and TVV3 (RRID:AB_2910121) capsid proteins (a kind gift from Dr. J-H. Tai, Academia Sinica, Taiwan) and Tsp1 (dilution of antibodies 1:1000). The goat anti-rabbit and anti-rat IgG-peroxidase antibodies (1:2000; Sigma-Aldrich, St. Louis, United States) were used as secondary antibodies. Proteins were visualized using chemiluminescence (Immobilon Forte, Merck, Kenilworth, United States), images were obtained with the Amersham Imager 600 (GE Healthcare, Chicago, Unites States), and signals were quantified using ImageJ/Fiji software (Schindelin et al., 2012).

### Multi-Angle Dynamic Light Scattering Analysis of Small Extracellular Vesicle Size

The hydrodynamic diameter of sEVs in selected fractions from the OptiPrep gradient was measured using a light scattering system with Malvern Zetasizer Ultra (Malverm Pananalytical Ltd., Malvern, United Kingdom). The measurement parameters were: size measurement mode – backscattering (173°), viscosity – water, temp –20°C. 50 μl of the sample was measured in a ZEN2112 quartz cuvette. Data analysis was performed by particles size distributed by number.

### Extracellular Vesicle Protection Assays

For protein protection assay, isolated sEVs were treated with trypsin (5 μg/ml) for 10 min at 37°C. In parallel, Triton X-100 was added to the final concentration of 0.1% to dissolve the sEV membrane before trypsin treatment. Treatment with trypsin was stopped by methanol/chloroform protein precipitation, and proteins were analyzed by immunoblotting. For RNA protection assay, samples of sEVs, untreated or treated with 0.1% Triton X-100, were incubated with RNAse A (10 mg/ml; Thermo Fisher, Waltham, MA, United States) for 10 min at 37°C. After the incubation, sEVs RNA was isolated using Trizol reagent (Merck, Darmstadt, Germany).

### Quantitative Mass Spectrometry

Label-free quantitative mass spectrometry (LFQ-MS) was performed as described previously (Zimmann et al., 2021). The samples were digested with trypsin and the peptides obtained were subjected to nano-liquid chromatography-mass spectrometry. The MS/MS spectra were searched against the *T. vaginalis* database (TrichDB, www.trichdb.org, release 2020-05-27, 60,330 entries), and 92 TVV protein sequences available in NCBI non-redundant protein database. The quantifications were performed with the label-free algorithms using MaxQuant LFQ version 1.6.2.0 (Max-Planck-Institute of Biochemistry, Planegg, Germany; Cox et al., 2014). The MS data were obtained from three independent sEV isolations and deposited to the ProteomeXchange Consortium *via* the PRIDE partner repository (Perez-Riverol et al., 2019) with the dataset identifier PXD031790.

### MS Data Processing

MaxQuant output file was processed using (Rstudio, Boston, MA, United States). To filter the initial dataset we used three criteria: we removed all single peptide identifications, all proteins identified in less than two independent experiments, and soluble proteins that were enriched in the supernatant after separation of sEVs with at least 1.5 fold change (*p*-value 0.02) in comparison to proteins in sEV fraction. To compare TVV plus and TVV minus sEV proteomes we conducted an imputation of all missing values (NA) with the normal distribution of the spectrometer limit and the final dataset was used to construct a volcano plot with a significance threshold *p*-value of 0.01 in RStudio.

### Bioinformatics

Each protein identified in the proteome of exosomes was annotated based on the TrichDB database (www.trichdb.org, release 52), and searched against the HMMs profiles of UniRef30_2020_06_hhsuite database (from http://gwdu111.gwdg.de/c̃ompbiol/uniclust/2020_06/) by hhblits version 3.3.0 (Steinegger et al., 2019). Conserved domains were predicted using the Pfam 34.0 database.^1^ The proteins were clustered based on assignment to Clusters of Orthologous Groups of proteins (COGs; Galperin et al., 2021) with the eggNOG-mapper v. 2.1.7 using the inbuilt eukaryotic database (Cantalapiedra et al., 2021). The presence of signal peptides was predicted using the TargetP version 2.0 (Emanuelsson et al., 2007). Transmembrane domains were predicted using the TMHMM server version 2.0 (Krogh et al., 2001).

### Real-Time Quantitative PCR

Total RNA was isolated from TV79-49c1^+^ and TV79-49c1^–^ using the Highpure RNA isolation kit (Roche Diagnostics, Mannheim, Germany) following the manufacturer’s protocol. The RNA was diluted to 50 ng/μl and 100 ng was used in each reaction for cDNA synthesis. RT-qPCR was performed using the Kapa SYBR FAST one-step Universal kit (Sigma-Aldrich, MI, United States). DNA topoisomerase II (TVAG_038880) was used for normalization as described (Narayanasamy et al., 2021). The primers are listed in Supplementary Table 1. All the data were analyzed in GraphPad Prism 7 (La Jolla, CA, United States) and the experiments were performed in triplicates.

### RNA Extraction From sEVs and Sequencing

RNA was extracted from sEVs using Trizol reagent and purified on the Zymo IIC spin column (RNA clean & Concentrator-25) that yielded 25–40 ng/μl of sEV RNA. Six libraries were prepared from three independent experiments (A, B, C), each with sEV RNA isolated in parallel from TV79-49c1^+^ and TV79-49c1^–^ cells. RNA integrity was checked using the RNA Nano 6000 Assay Kit of the Bioanalyzer 2100 system (Agilent Technologies, Santa Clara, CA, United States), and concentration was determined with Qubit^®^ RNA Assay Kit in Qubit^®^ 2.0 Fluorometer (Life Technologies, Carlsbad, CA, United States). Small RNA-Seq libraries were prepared from 200 ng of RNA using the NEXTFLEX^®^ Small RNA-Seq Kit v3 for Illumina^®^ Platforms (Perkin Elmer, Waltham, Unites States). Libraries were pooled in equimolar amounts, a 9 pM solution was loaded on the Illumina sequencer MiSeq (Illumina, San Diego, CA, United States) and sequenced uni-directionally, generating 50 bases long reads. Library preparation and sequencing were performed at the GeneCore facility of the European Molecular Biology Laboratory, Heidelberg, Germany.

### Reverse Transcription PCR

The sEVs RNA (200 ng) was used for cDNA synthesis with the Verso cDNA synthesis kit (Thermo Fisher Scientific, Waltham, MA, United States) following the manufacturer’s instruction. The cDNA was used as a template for PCR, which was performed with Phusion Green Hotstart II High-Fidelity PCR Mastermix (Thermo Fisher Scientific, Waltham, MA, United States). The following cycling conditions were applied: Initial denaturation at 98°C for 2 min, 30 cycles of denaturation 98°C/10 s, 60°C/30 s, 72°C/45 s, and final extension at 72°C for 5 min. The PCR amplicons were separated on 1% agarose gel and the images were captured in InGenius3 (Syngene, Cambridge, UK).

### RNA Data Processing

Cutadapt v1.8.3 (https://cutadapt.readthedocs.io/en/v1.9.1/) was used to remove the adapter (TGGAATTCTCGGGTGCCAAGG), first 4 nt and last 4 nt, and to filter out sequences < 15 nt. Sequences were normalized per million reads for comparison between experiments. RNA fragments were mapped to reference TVV genomes sequences TVV1 NC_027701.1, TVV2 NC_003873.1, TVV3 NC_004034.1, and TVV4 NC_038700.1, and a reference genome of *T. vaginalis* at TrichoDB (Amos et al., 2022; TrichDB-52_TvaginalisG3.gff) using Bowtie2 version 2.4.4 with end-to-end alignment option (Langmead and Salzberg, 2012). The mapped reads were assigned to their respective genes using FeatureCounts. Differential expression analysis was conducted using DESeg2 package in Rstudio. To create the bar charts and pie charts of RNA and tRNA distribution, genes were assigned to different groups according to their Trich-DB annotation. All reads and filtered tRNA reads were grouped by their lengths to create the length distribution graphs. To calculate the coverage and distribution of different tRNAs, coverageBed version 2.30.0 (https://bedtools.readthedocs.io/en/latest/content/tools/coverage.html) was used.

Sequences were deposited in the European Nucleotide Archive, under accession number PRJEB50674.

### Electron Microscopy

The negative staining of sEV was performed using 5 μl of a sample that was incubated on glow discharged carbon-coated copper grid for 5 min. The grid was then washed in Milli-Q-H_2_O and stained with 2% uranyl acetate. The samples were observed in a transmission electron microscope (TEM) JEOL JEM 2100Plus (Akishima, Japan) equipped with an XF416 CMOS camera (TVIPS GmbH, Gauting, Germany) at 200 kV. For the cryoEM analysis, the samples were prepared using Automatic Plunge Freezer EM GP2 (Leica Microsystems GmbH, Wetzlar, Germany). The sample (4 μl) was placed on the glow discharged Quantifoil^®^ carbon grid (R 1.2/1.3), blotted with filter paper for 4 s, and directly plunged in liquid ethane tempered to –143°C with liquid nitrogen. The JEOL JEM-2100Plus, at 200 kV was used for the analysis under cryo conditions with Gatan 626 cryo holder (Gatan, Inc., Pleasanton, CA, United States) and SerialEM software version 3.9 beta1 (https://bio3d.colorado.edu/SerialEM/).

### Expression of BspA in Trichomonads and Immunofluorescence Microscopy

Gene for BspA (TVAG_268070) was amplified by PCR from *T. vaginalis* genomic DNA and cloned into plasmid TagVag for episomal expression with C-terminal hemagglutinin tag in *T. vaginalis*. Trichomonads were transformed and selected as described (Hrdy et al., 2004). Immunofluorescent microscopy was performed as described (Stáfková et al., 2018). BspA was detected in trichomonads by the combination of mouse monoclonal antibody against 2x-HA tag (Exbio, Czechia), and secondary donkey anti-mouse antibody Alexa Fluor 488 (Thermo Fisher Scientific, Waltham, MA, United States). Structured illumination microscopy (SIM) was performed as described (Stáfková et al., 2018).

### TVV Transmission Experiments

EVs were isolated from TV79-49c1^+^ as described above. The equivalent of EVs produced by 1.5 × 10^8^ Tv79-49c1^+^ cells (5 mg of protein) was added to the suspension of 1.5 × 10^5^ TV79-49c1^–^ cells per ml (total volume of 10 ml of TYM media). Trichomonads with microvesicles were incubated in TYM medium for 24 h to reach cell density 1.5 × 10^6^ per ml, then 1 × 10^6^ cells were transferred to 10 ml of the fresh medium, and 9 × 10^6^ cells were harvested and used for RNA isolation. The same procedure was performed for consecutive four subcultures.

To test the TVV transmission between trichomonads, we prepared two cell lines. The donor line was TV79-49c1^+^ that was transformed with pTagVagV5-Pur (Rada et al., 2019), enabling the expression of biotin ligase (BirA) as a marker under puromycin selection (TV79-49c1^+^ PAC-BirA). The recipient strain was TV79-49c1^–^ that was transfected with pTagVag2 (Rada et al., 2015) for expression of cytosolic HA-tagged phosphofructokinase under geneticin selection (TV79-49c1^–^ G418-PFK). Both trichomonad strains were mixed based on their doubling times (1.1 × 10^5^ of TV79-49c1^+^cells and 5.3 × 10^4^ of TV79-49c1^–^cells per ml) to reach the density of 1.5 × 10^6^ cells per ml after 24 h of co-cultivation. After five transfers, geneticin was added to the culture (200 μg/ml) to eliminate the TVV donor strain, and the selection with geneticin was maintained for 40 transfers. Total RNA was isolated at selected time points from washed cells for RT PCR analysis as above.

### Immune Biomarkers Determination

HaCaT cells were grown to 80-90% confluency in RPMI 1640 medium in a total volume of 5 ml. Subsequently, the cells were incubated in fresh RPMI 1640 medium with the sEVs and at 37°C for 24 h in the 5% CO_2_ atmosphere. The treatment dose of sEVs was calculated to be equivalent to a load of parasites during vaginal infection (4 × 10^5^ trichomonads/ml, 27 μg sEVs per 1 ml HaCaT cell culture). After incubation, the conditioned medium was centrifuged for 10 min at 1000 × *g* and 4°C, divided into aliquots, and stored at –80°C. The isolations of sEVs were performed in triplicate for each cell line (TV79-49c1^±^) and each batch of harvested sEVs was co-incubated with technical triplicate of HaCaT cells. Unstimulated HaCaT cells conditioned medium was used as a negative control.

Levels of selected immune mediators (IL-6, IL-8, RANTES, CCL-2, and IL-1β) were analyzed by ELISA kits, performed in triplicates according to the manufacturer’s instructions (Thermo Fisher Scientific, Waltham, Unites States).

## Results

### *Trichomonas vaginalis* Exosomes Contain TVV Particles

**FIGURE 1:**
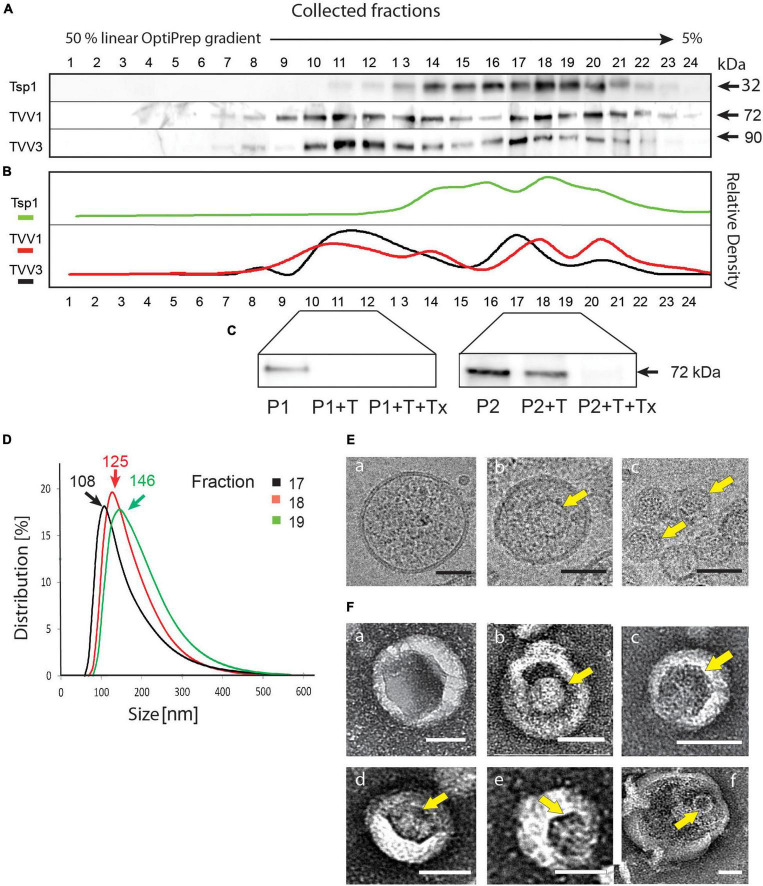
Small EVs produced by *Trichomonas vaginalis* TV79-49c1^+^ contain *Trichomonasvirus* (TVV) particles. **(A)** Immunoblot analysis of 24 fractions collected from the last step of EV separation using linear OptiPrep gradient. Antibodies against Tsp1 (sEV marker), and TVV1 and TVV3 capsid proteins were used for screening of fractions. **(B)** Densitometry of western blots. **(C)** Protein protection assay. Membrane protection of TVV proteins was monitored by immunoblotting using antibodies against TVV1 capsid protein. P1, the pool of fractions 10–12; P2, the pool of fractions 17–19; T, trypsin; Tx, Triton X100. **(D)** Multi-angle dynamic light scattering analysis of sEV size in fractions 17, 18, and 19. **(E)** (a–c) Cryo-electron microscopy of sEV fraction. **(F)** (a–f). Electron microscopy of sEVs after negative staining. Arrows point to TVV particles. Bars indicate 40 nm.

To test the hypothesis that TVV is exported from *T. vaginalis* cells *via* EVs, we isolated EVs from the clone TV79-49c1^+^ that harbors TVV1, TVV2, and TVV3 (Narayanasamy et al., 2021). EVs were isolated based on the combination of filtration and centrifugation of conditioned medium using a linear gradient of 5–50% OptiPrep in the final step. For monitoring of sEVs in the gradient fractions, we developed a polyclonal antibody against tetraspanin Tsp1 (TVAG_019180) that has been identified in the proteome of *T. vaginalis* exosomes (Twu et al., 2013). The major signal on the western blot for Tsp1 was observed in fractions 17–19 of 24 collected fractions (Figures 1A,B). Dynamic light scattering analysis of isolated EVs in fractions 17–19 revealed peak sizes 108–146 nm (Figure 1D; hereafter sEVs), which is in the range of *T. vaginalis* exosomes (Twu et al., 2013). The presence of TVVs was monitored by antibodies against the capsid protein of TVV1 (Liu et al., 1998) and TVV3 (Bessarab et al., 2011; Narayanasamy et al., 2021). The maximal signals for both TVVs were in the fractions enriched in sEVs, however, there was another maximum in more dense fractions (10–12; Figures 1A,B). We assumed that in the sEV fractions, the viral particles are inside of the vesicles whereas the second maximum may represent naked particles outside of sEVs. To test the TVV topology, we treated pooled sEV fractions (17–19), and more dense fractions (10–12) with trypsin (Figure 1C). The trypsin treatment had a minor effect on the capsid protein signal in the exosome fractions; the capsid protein disappeared only upon the addition of the membrane-dissolving detergent Triton X-100. The capsid protein in the more dense sample (fractions 10–12) was not membrane-protected and was cleaved without adding the detergent. Next, we used sEV fractions for analysis by cryo-electron microscopy which is optimal to observe vesicles in shape close to their physiological stage. The sEVs appeared as spherical vesicles surrounded by a single membrane of 122 ± 20 nm (*n* = 20) in diameter (Figure 1Ea). In some vesicles we observed TVV particles of about 40 nm (Figure 1Eb), infrequently the viral particles were detected also outside of exosomes (Figure 1Ec). Similarly, we found individual TVV particles enclosed in vesicles of expected size using negative staining (Figure 1Fa–e), rarely do we find larger vesicles surrounding 2–3 viral particles (Figure 1Ff). Altogether, these results indicated that TVV particles resided inside of sEVs released by *T. vaginalis* TV79-49c1^+^.

### TV79-49c1^–^ Failed to Be Re-infected With TVV

**FIGURE 2:**
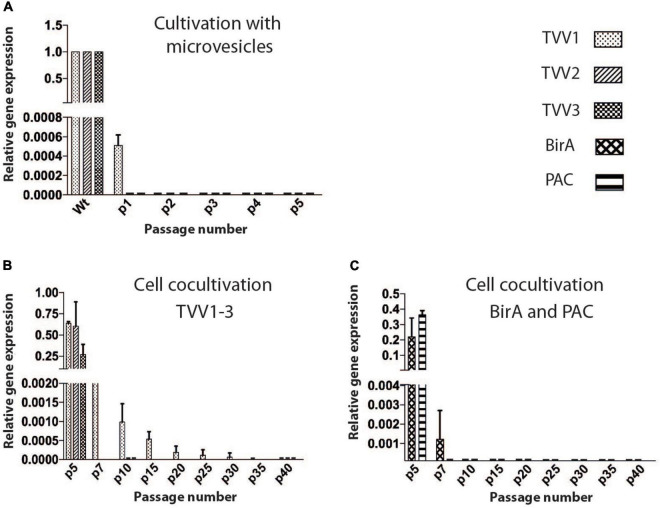
TVV transmission experiments. **(A)** Monitoring of TVVs by RT PCR in TV79-49c1^–^ clone after incubation with sEVs from TV79-49c1^+^. **(B)** RT PCR monitoring of TVVs, in TV79-49c1^–^ acceptor clone for 40 subcultures after co-cultivation with TVV79-49c1^+^ donor clone. TVV- plus clone was transformed with a plasmid for expression of puromycin N-acetyl transferase (PAC), and biotin ligase (BirA), and the TVV-minus clone was resistant to geneticin. The clones were mixed, co-cultivated for five subcultures, and then geneticin was added for 35 subcultures. **(C)** RT PCR monitoring of genes for BirA and PAC.

Identification of TVV particles in sEVs prompted us to test whether the sEVs may serve for transmission of TVV to TVV-free *T. vaginalis*. Initially, we isolated EVs from the TV79-49c1^+^ clone and added access (equivalent that produces 1.5 × 10^8^ of Tv79-49c1^+^) to the culture of TV79-49c1^–^ (1.5 × 10^6^ cells in 10 ml). Like in Tv79-49c1^+^ cells, all three TVVs were detected in the isolated EVs by RT PCR (Supplementary Figure 1). However, a negligible amount of TVV1 and no TVV2 and TVV3 were detected in *T. vaginalis* cells after 24 h incubation with TVV positive EVs. The cells in the following four subcultures were negative for all three TVVs (Figure 2A and Supplementary Figure 1).

Next, we rationalized that isolation of EVs may affect their biological properties, and interfere with TVV transmission, thus we decided to directly co-cultivate TVV plus and TVV minus trichomonad clones. For this purpose, we derived the donor TV79-49c1^+^ clone, which was transformed with pTagVagV5-Pur (Rada et al., 2019), expressing a puromycin N-acetyl-transferase (PAC) for puromycin resistance and biotin ligase (BirA) as a marker (TV79-49c1^+^ PAC-BirA). The recipient strain TV79-49c1^–^ was transformed with pTagVag2 (Rada et al., 2015), providing resistance to geneticin (TV79-49c1^–^ G418-PFK). The clones were mixed, co-cultivated for five subcultures, and then geneticin was added to ablate the donor trichomonads. RT PCR signal for *pac* disappeared after two subcultures with geneticin, and neither *pac* nor *birA* was detectable after five subcultures indicating an absence of the donor trichomonads (Figures 2B,C and Supplementary Figure 2). TVV1 signal gradually declined, but it was still detectable after 30 subcultures and disappeared after 35 subcultures. Thus, the co-cultivation did not lead to the stable TVV infection of TVV-free clones under our experimental conditions.

### Effect of TVV Infection on sEV Proteome

**FIGURE 3:**
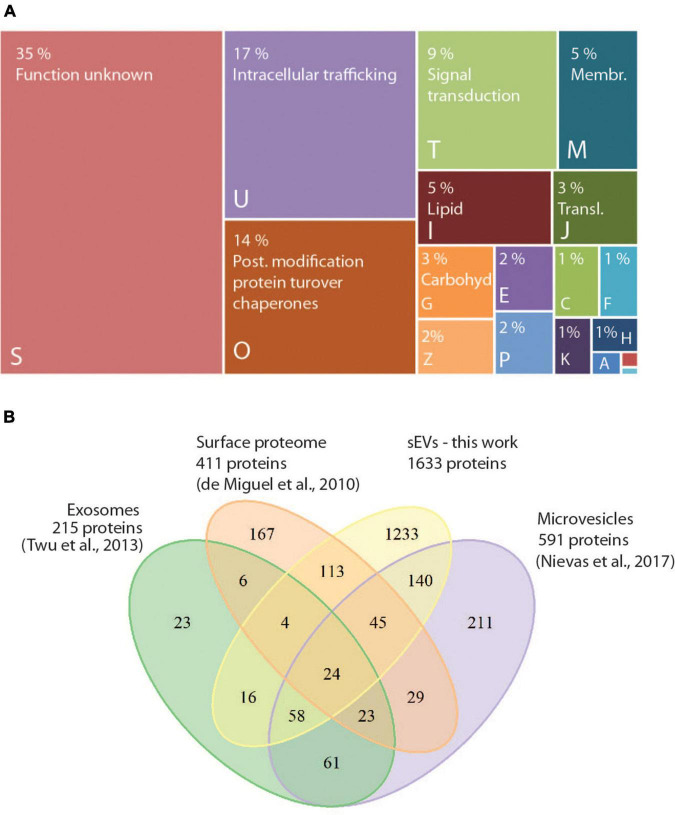
Assignment of protein in the proteome of sEVs to functional categories. **(A)** COG (Clusters of orthologous groups) functional categories of identified proteins in sEVs from *T. vaginalis* TV79-49c1^+^ and TV79-49c1^–^. Capital letters correspond to COG categories: A, RNA processing and modification; B, Chromatin structure and dynamics; C, Energy production and conversion; E, Amino acid transport and metabolism; F, Nucleotide transport and metabolism; G, Carbohydrate transport and metabolism; H, Coenzyme transport and metabolism; I, Lipid transport and metabolism; -J, Translation, ribosomal structure and biogenesis; K, Transcription; M, Cell wall, membrane, envelope biogenesis; O, Posttranslational modification, protein turnover, chaperones; P, Inorganic ion transport and metabolism; S, unknown function; T, Signal transduction mechanism; U, Intracellular trafficking, secretion, and vesicular transport; Z, Cytoskeleton. **(B)** Venn diagram comparing proteomes of sEVs, exosomes (Twu et al., 2013), surface proteome (De Miguel et al., 2010), and microvesicles (Nievas et al., 2018).

To investigate whether TVV infection modifies exosomal cargo, we first compared proteomes of exosomes produced by isogenic *T. vaginalis* clones TV79-49c1^+^, and its virus-free derivative TV79-49c1^–^. The size of sEVs produced by TV79-49c1^–^ was similar to those from TV79-49c1^+^ (113–150 nm, Supplementary Figure 3), and both isogenic clones produced a comparable amount of sEVs, estimated as the production of sEV proteins. The TV79-49c1^+^, and TV79-49c1^–^ clones released sEVs corresponding to 13.02 ± 1.29 μg, and 14 ± 0,69 μg (*n* = 3) of sEV proteins per 1 h by 1 × 10^6^ cells. In total, we identified 1633 proteins including conserved exosomal proteins such as Tsp1 and five other tetraspanins, Rab small GTPases (146 proteins), Hsp70 (20 proteins), Tsg101, and cytoskeletal proteins actin (7 paralogs), and tubulin (Supplementary Table 2). The proteins were classified based on clusters of orthologous groups (COGs) into 18 functional categories (Figure 3A). The largest functional category formed proteins that are involved in intracellular trafficking (COG category U, 17%), namely SNARE proteins (putative synaptobrevins, syntaxins), vacuolar protein sorting-associated proteins (Vps), and multiple Rab paralogs of twelve Rab families, as well as unclassified Rabs. The most prominent group of proteins in the COG category “posttranslational modification, protein turnover, chaperones” (COG O, 14%) were peptidases (65 in total). Of these, membrane-associated peptidases included serine peptidases (10 paralogs), two calcium-dependent cysteine proteases (calpains), and leishmanolysin-like metallopeptidases (GP63, 11 paralogs), while the most frequent soluble peptidases were cysteine peptidases (CPs, 18 proteins) of various families, including legumains, calpain-like CPs, ubiquitin-hydrolase-like CPs, and metacaspases. The third-largest COG category included proteins for signal transduction mechanism (COG T, 9%) with 35 protein kinases, 13 proteins with predicted phosphatase activity, and 15 calcium-binding proteins including 7 calmodulin paralogs (Supplementary Table 2). Our dataset was considerably larger in comparison to the previously reported exosomal proteome of 215 proteins, of which 47% were shared with our dataset (Figure 3B). Comparison with the proteome of microvesicles shed from *T. vaginalis* plasma membrane (ectosomes; Nievas et al., 2018) and the surface proteome (De Miguel et al., 2010) revealed 45 and 43% common proteins, respectively (Figure 3B).

**FIGURE 4:**
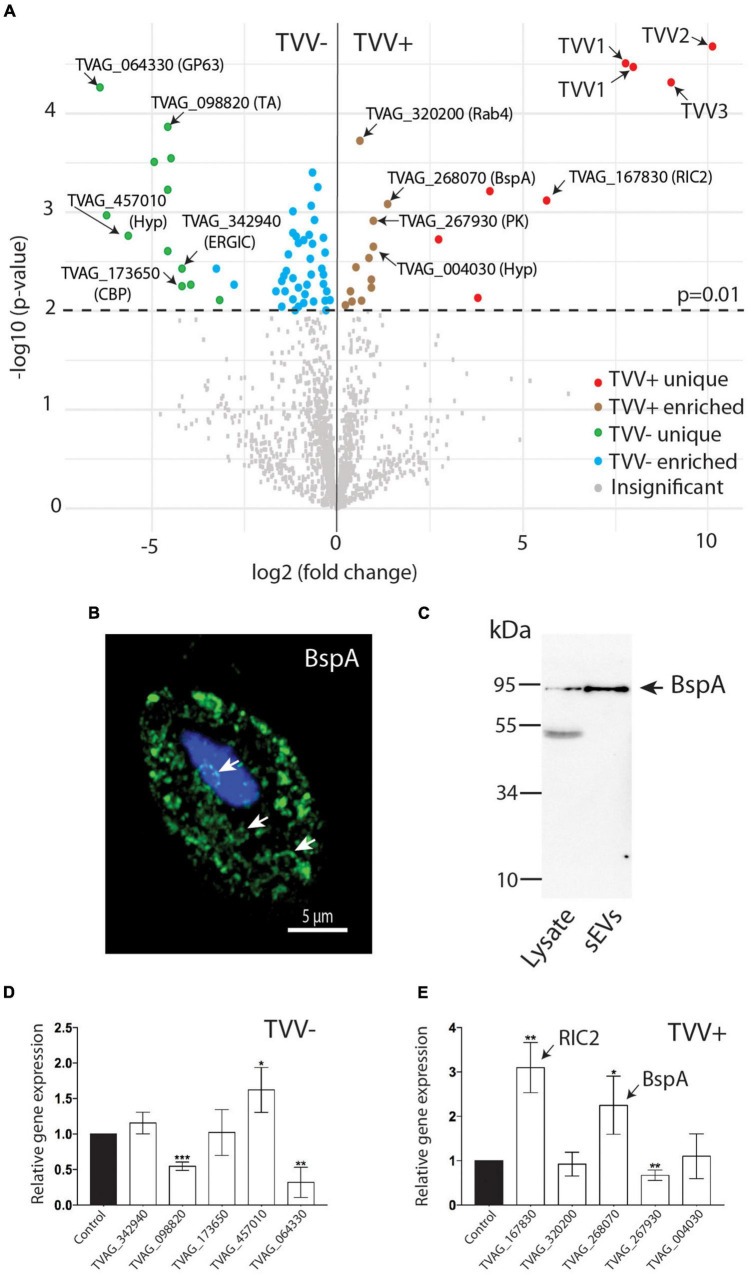
Comparison of sEVs proteomes from TV79-49c1^–^ (TVV–) and TV79-49c1^+^(TVV +). **(A)** Volcano plot analysis of proteomes using statistical significance *p*-value 0.01. **(B)** Structured illumination microscopy of *T. vaginalis* TV79-49c1^+^ expressing HA-tagged BspA (TVAG_268070). Arrows indicate large vesicles reminiscent of multivesicular bodies. **(C)** Immunoblot detection of HA-tagged BspA (TVAG_268070) in cell lysate and sEVs from TV79-49c1^+^ transformed *T. vaginalis*. BspA corresponds to 87 kDa protein in the cell lysate and sEVs. An additional band of 50 kDa in the lysate is likely a product of BspA cleavage. D, E. RT PCR of selected genes for proteins, which were unique or upregulated in proteomes of TVV– **(D)** or TVV+ **(E)** sEVs. CBP, calcium-binding protein; ERGIC, endoplasmic reticulum-Golgi intermediate compartment protein; GP63, leishmanolysin-like metallopeptidase; hyp, conserved hypothetical protein; PK, CMGC family protein kinase; RIC2, repair of iron centers protein 2; TA, tyrosine aminotransferase. *p-value < 0.05, **p-value < 0.005, ***p-value = 0.0005.

Quantitative analysis of differentially expressed proteins in TV79-49c1^+^and TV79-49c1^–^ sEVs with a statistical cut-off *p*-value of 0.01 revealed 12 significantly enriched and 8 unique proteins in TVV plus sEVs (Figure 4A and Supplementary Table 2). The unique proteins that were absent in TV79-49c1^–^ sEVs included four TVV capsid-RdRp fusion proteins. Mapping of individual peptides against 92 available protein sequences of TVVs revealed that the majority mapped to capsid protein of TVV1 (21 peptides), 13 peptides to TVV2, 13 peptides to TVV3, and a single peptide corresponded to RdRp of TVV1 (Supplementary Table 3). The protein sequence alignment also revealed the presence of unique peptides that mapped to different variants of TVV species: four variants of TVV1, and two variants of each TVV2 and TVV3 species (Supplementary Figure 4). The other unique proteins in TV79-49c1^+^ sEVs included the repair of iron centers protein 2 (RIC2, TVAG_167830; Nobre et al., 2016), a membrane protein TVAG_109130 with N-terminal subtilisin-like peptidase (IPR036852), and galactose-like binding (IPR008979) domains, and two proteins of unknown function (Figure 4A). The enriched proteins included putative adhesin BspA (TVAG_268070) with the C-terminal transmembrane domain (TMD). This protein is of particular interest as exosomes have been shown to modulate *T. vaginalis* adherence (Twu et al., 2013). In addition to TVAG_268070, the proteome contains 18 other BspA-like paralogs, however, these were not significantly enriched in TVV plus sEVs (Supplementary Table 2). To verify cellular localization of BspA TVAG_268070, the recombinant protein with the C-terminal HA tag was expressed in TV79-49c1^+^. Structural illumination microscopy showed the presence of BspA in the membrane of large vesicles reminiscent of multivesicular bodies (Figure 4B; Twu et al., 2013). Western blot analysis showed a band corresponding to BspA of the expected size (87 kDa) in cell lysate as well as in shed sEVs (Figure 4C). The other enriched proteins included discoidin-like adhesin (TVAG_004030), putative calcium-dependent phospholipid-binding protein (TVAG_069450), Rabs (TVAG_320200, TVAG_044270), protein kinase domain-containing protein (TVAG_037610), vacuolar V-ATPase subunit B and C (TVAG_037610, TVAG_324980), a metallopeptidase M24 (TVAG_410220), and three unknown proteins. The V-ATPase is a multisubunit complex, thus we searched for other subunits insEV proteome. We identified ten additional proteins corresponding to ATPase subunits B, C, D, E, and H, of which nine were enriched in TV79-49c1^+^sEVs at *p*-value ≤ 0.05. The proteome of TV79-49c1^–^ sEVs revealed 12 unique and 42 enriched proteins. Of these, the largest functional groups were formed by proteins involved in intracellular trafficking and exocytosis (11 proteins, 20%), and hydrolases (8 proteins, 15%) including three GP63 peptidases and alpha-1,3-glucosidase. The group of proteins with unknown functions included 17 members (31%; Figure 4A and Supplementary Table 2).

To investigate whether the increased level of proteins in sEVs correlates with the level of the corresponding mRNA in *T. vaginalis* cells, we isolated total RNA from TV79-49c1^+^and TV79-49c1^–^, and quantified RNA using qRT PCR for five proteins enriched or unique in TVV plus and TVV minus sEVs. This analysis revealed over 2-fold higher RNA level in TV79-49c1^+^ for *RIC2* and *BspA* in comparison to TV79-49c1^–^, which correlated with a higher level of corresponding proteins, however, no significant difference or decrease was found for TVAG_320200, TVAG_267930, and TVAG_004030 (Figure 4D). Similarly, a limited correlation between protein and mRNA levels was observed for five selected proteins that were unique in TV79-49c1^–^ (Figure 4E). Particularly, TVAG_064330 (GP63) and TVAG_098820 displayed significantly lower mRNA levels in TV79-49c1^–^ in comparison to TV79-49c1^+^ although these proteins were present only in TV79-49c1^–^ sEVs (Figure 4A). The positive correlation between mRNA and protein levels for RIC2 and BspA suggested that TVV infection may have an impact on gene transcription or mRNA stabilization. The higher mRNA level with the absence of corresponding proteins (GP63, and TVAG_098820) in TV79-49c1^+^ suggested that TVV infection may interfere with the protein translation or packaging of the exosomal cargo in *T. vaginalis* cells, however, the proposed explanation of observed phenomena needs experimental verification.

### TVVs Modulate Small RNA Cargo in EVs

**FIGURE 5:**
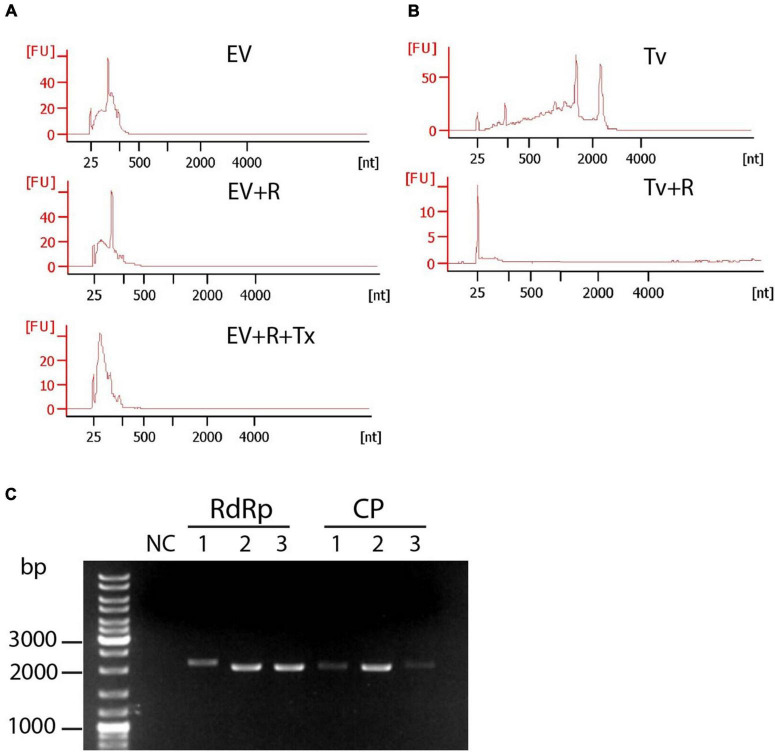
Protection assay of sRNAs in sEVs of TV79-49c1^+^. **(A)** Small RNAs were treated with RNAse A (EV + R) or RNAse A and Triton X-100 (EV + R + Tx). Arrows point to a prominent peak of 100 nt. **(B)** Treatment of *T. vaginalis* cellular RNAs (Tv) with RNAse A (Tv + R). RNA was analyzed using Bioanalyzer. **(C)** Amplification of ORFs for RdRp and capsid protein (CP) of TVV1, TVV2, and TVV3 from RNA isolated from sEVs.

To investigate whether TV79-49c1^+^sEVs contain TVV-derived RNA and how the presence of TVV modulates sEV RNA cargo, aliquotes of exosomal samples from TV79-49c1^+^ and TV79-49c1^–^ isolated for proteomic analysis were in parallel used for RNA isolation (Figure 5A RNA analysis). The majority of isolated RNAs appeared to be of a small size ranging between 25 and 200 nt (Figure 5B). We did not observe a signal corresponding to the size of TVV genomes of 4.6–4.8 kbp, however, we presumed that the amount of complete genomic RNA was under the limit of electrophoretic detection or only fragments of genomes were present. Indeed, we amplified from isolated RNA complete ORFs for RdRp from TVV1, 2, and 3 (2037, 2130, and 2127 bp), and for capsid proteins (2205, 2106, and 2046 bp; Figure 5C) by reverse transcription PCR. To test whether sEVs-associated RNA is membrane-protected, we treated EVs with RNase A before RNA isolation, which had a negligible effect on RNA sizes. The addition of Triton X-100 together with RNase resulted in partial RNA digestion, particularly the disappearance of the dominant peak of approximately 100 nt (Figure 5B). As an unprotected control, we isolated cellular RNA from *T. vaginalis* with characteristic peaks of ribosomal RNAs on the electrophoretogram that disappeared upon treatment with RNase A without the detergent addition (Figure 5C). These results confirmed that exosomal RNAs were protected by a membrane envelope.

In total, six RNA-seq libraries were prepared from three independent experiments. Sequencing of each library generated on average 2.8 million reads per library, each read 50 nt long. The total number of filtered reads ranged from 1.8 to 3.9 million (Table 1). Mapping of reads to reference TVV1-4 genomes revealed 248-6029 reads in TV79-49c1^+^sEVs cargo matching TVV1, TVV2, and TVV3 genomes. The reads were distributed along the whole genome sequence covering 45.7–82.1% of the genome sequences (Supplementary Figure 5). A negligible number of reads (1–11) derived from TV79-49c1^–^ EVs library randomly mapped to TVVs.

**TABLE 1:** Sequencing of RNAs isolated from sEVs that were shed by TV79-49c1^–^, and TV79-49c1^+^.

Sample*	A: TVV^–^	A: TVV^+^	B: TVV^–^	B: TVV^+^	C: TVV^–^	C: TVV^+^
Total read number (<15 bp)	1,844,606	1,827,363	2,417,076	3,916,028	3,644,654	2,905,518
Reference dataset TVV1-4**	4	4	4	4	4	4
Mapped reads TVV1, (% coverage)	2	783 (45.7)	0	2880 (53.6)	0	1202 (47.4)
Mapped reads TVV2 (% coverage)	4	248 (50.9)	0	1480 (63.9)	1	1059 (61.6)
Mapped reads TVV3 (% coverage)	11	781 (56.3]	1	6029 (86.2)	0	3094 (82.1)
Mapped reads TVV4	0	1	0	1	0	0
Reference transcript TV G3^#^	98,611	98,611	98,611	98,611	98,611	98,611
Total mapped reads(%)^##^	1,046,850 (56,75)	952,694 (52.13)	1,310,931 (54.24)	2,095,038 (53.5)	1,910,888 (52.43)	1,717,371 (59.11)
Unmapped (%)	797,756 (43.25)	874,669 (47.87)	1,106,145 (45.76)	1,820,990 (46.50)	1,733,766 (47.57)	1,188,147 (40.89)
tRNA RPM (%)^†^	143,009 (14.3)	415,034 (41.5)	102,327 (10.23)	161,381 (16.14)	83,516 (8.35)	232,954 (23.3)
rRNA RPM (%)^†^	841 868 (84.19)	558 347 (55.83)	883 470 (88.35)	814 944 (81.49)	902,531 (90.25)	748,027 (74.8)
Others RPM (%)^†^	15,123 (1.51)	26,619 (2.66)	14,203 (1.42)	23,675 (2.37)	13,953 (1.4)	19,019 (1.9)

*RNA was isolated from three independent experiments (A, B, and C), each experiment included isolation of RNA cargo from TV79-49c1^–^ (TVV^–^), and TV79-49c1^+^ (TVV^+^) sEVs.

**Reference dataset: TVV1 (NC_027701.1), TVV2 (NC_003873.1), TVV3 (NC_004034.1), and TVV4 (NC_038700.1).

*^#^Reference genome: T. vaginalis G3 strain, TrichoDB (https://trichdb.org/trichdb/app).*

*^##^end-to-end (bowtie2, http://bowtie-bio.sourceforge.net/bowtie2/index.shtml).*

*^†^FeatureCounts has been used for assignment.*

*RPM, read per million, (%) percent of assigned reads.*

**FIGURE 6:**
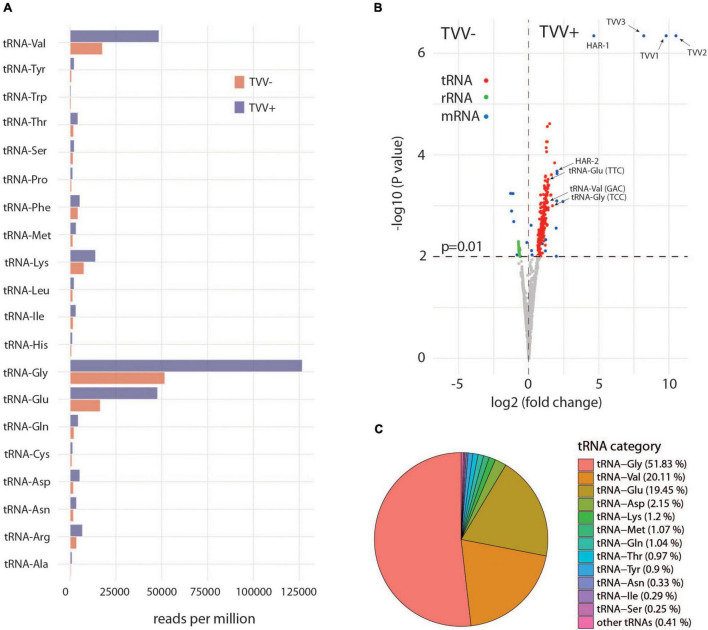
Transfer RNAs are enriched in TV79-49c1^+^. **(A)** Distribution of tRNA types in sEVs from TV79-49c1^–^ (TVV-), and TV79-49c1^+^ (TVV +). **(B)** Volcano plot analysis of tRNAs, rRNAs, and mRNAs in TVV- and TVV+ sEVs. **(C)** Distribution of tRNA types that were significantly enriched in TVV+ sEVs in comparison to TVV- sEVs.

Next, the reads were mapped to reference *T. vaginalis* G3 genome (Table 1 and Supplementary Table 4). The majority of mapped reads were assigned to rRNA with a higher contribution in TV79-49c1^–^sEVs (on average 89%) than in TV79-49c1^+^ sEVs (70%; Table 1). The second group included tRNA with about a 2.5-fold higher number of total tRNA reads in TV79-49c1^+^ (on average 10%) than in TV79-49c1^–^ sEVs (27%; Table 1). Other gene coding sequences (CDS) represented on average 1.4% in TVV minus and 2.3% in TVV plus sEVs. Unmapped reads accounted for 41–48% in total (Table 1 and Supplementary Table 4). Mapping of tRNA reads revealed the presence of twenty tRNA types in both TVV plus and TVV minus sEVs with the majority of RNA reads mapping to tRNA-Gly, tRNA-Val, and tRNA-Glu (Figure 6A). The other tRNA types were represented in less than 2% each. Statistical analysis of tRNAs, rRNAs, and CDS RNAs with a p-value of 0.01 showed significant enrichment of tRNAs in TV79-49c1^+^ sEVs (Figure 6B). The most frequent type of enriched tRNA fragments corresponded to tRNA-Gly (51.8% of enriched tRNA fragments), tRNA-Val (20.1%), and tRNA-Glu (19.5%; Figure 6C). The other RNA fragments enriched in TV79-49c1^+^ sEVs corresponded to sixteen mRNAs with the most enriched fragments mapping to two genes for putative hydroxylamine reductases (HAR1-2). The unique fragments belonged to TVV RNAs in TV79-49c1^+^(Figure 6B and Supplementary Table 4).

**FIGURE 7:**
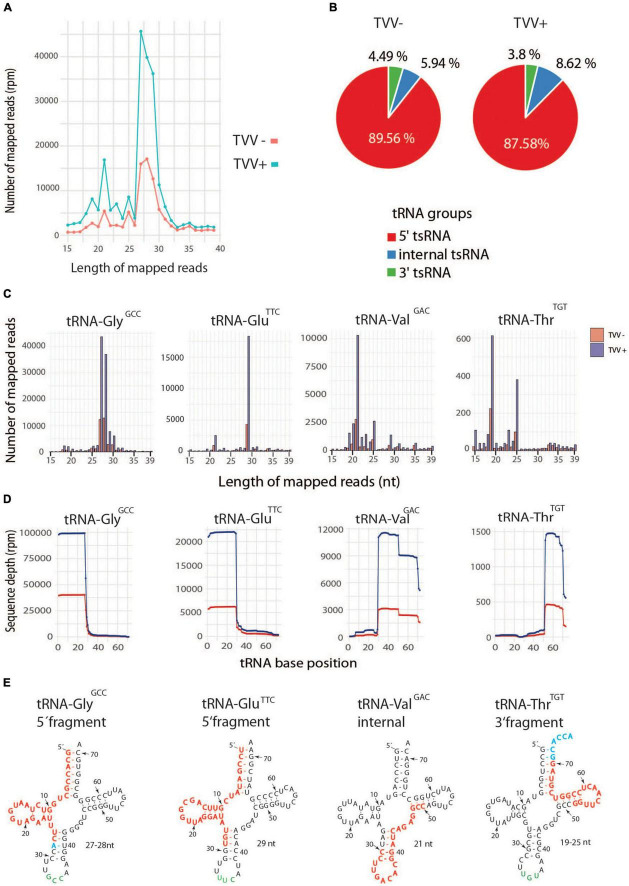
Enrichment of tsRNA in TVV + sEVs. **(A)** Size distribution of total tRNA fragments in TVV-, and TVV+ sEVs. **(B)** Distribution of fragment categories in TVV + sEVs. **(C)** Size distribution of the most frequent tsRNAs in TVV- and TVV+ sEVs. **(D)** Coverage of dominant tsRNAs that were mapped to corresponding tRNAs. Transfer sRNAs were grouped based on tRNA type and anticodon. **(E)** Mapping of most frequent tsRNA fragments to mature tRNAs. Red letters indicate a sequence of tsRNA, blue letters indicate extension in some sequences.

Transfer RNA fragments (tsRNA) are of particular interest as they belong to the emerging category of non-coding regulatory RNAs that are conserved in all domains of life including *T. vaginalis* (Li et al., 2018). They are sorted according to the tRNA region, from which they are derived by cleavage within T-loop, D-loop, or anticodon-loop (Dou et al., 2019). Thus, we sorted tsRNAs based on their gene mapping, which revealed two major peaks of tsRNAs with sizes of ∼ 21 nt and ∼ 27–29 nt with the higher number of all fragments in TV79-49c1^+^ sEVs (Figure 7A). In TV79-49c1^+^ sEVs, majority of tsRNA (87.6%) were classified as 5′tsRNA, 8.6% mapped to the middle of tRNA including anticodons region (internal tsRNA), and 3.8% were 3′tsRNAs (Figure 7B). A similar distribution but a lower quantity of tRNA fragments were found in TV79-49c1^–^ sEVs. For detailed classification, we grouped tsRNA based on tRNA type and anticodons (Figures 7C–E, Supplementary Figures 6, 7 and Supplementary Table 5). The most dominant tsRNAs in TV79-49c1^–^ sEVs were 5′tsRNA-Gly with GCC anticodon (58%), 5′tsRNA-Glu with TCC anticodon(13%), and internal tsRNA-Val with GAC anticodon(7%; Supplementary Table 5). In TV79-49c1^+^, the pattern of tsRNA was similar with a slightly higher contribution of 5′tsRNA-Gly (GCC; 60%), and a lower number of 5′tsRNA-Glu (TTC; 9%), and internal tsRNA-Val (GAC; 5%; Supplementary Table 5). Collectively, a common pattern of tsRNA in TVV plus and minus exosomes indicated that tsRNA biogenesis operates similarly in both cell lines, however, the tsRNA content is significantly enhanced in exosomes produced by TV79-49c1^+^, which suggests more efficient packaging of tsRNA cargo into exosomes upon TVV infection.

Unlike for tsRNA, mapping of fragments derived from 16S rRNA and 28S rRNA revealed even distribution along with the complete genes without enrichment of specific fragments. This distribution is more consistent with rRNA degradation than with the packaging of functional fragments (Supplementary Figure 8A). Interestingly, fragments derived from 5.8 rRNA appear to be 28 and 42 nt long (Supplementary Figure 8B–D), which suggests their specific processing.

### Small EVs Derived From TV79-49c1^+^ Activate Pro-Inflammatory Response

**FIGURE 8:**
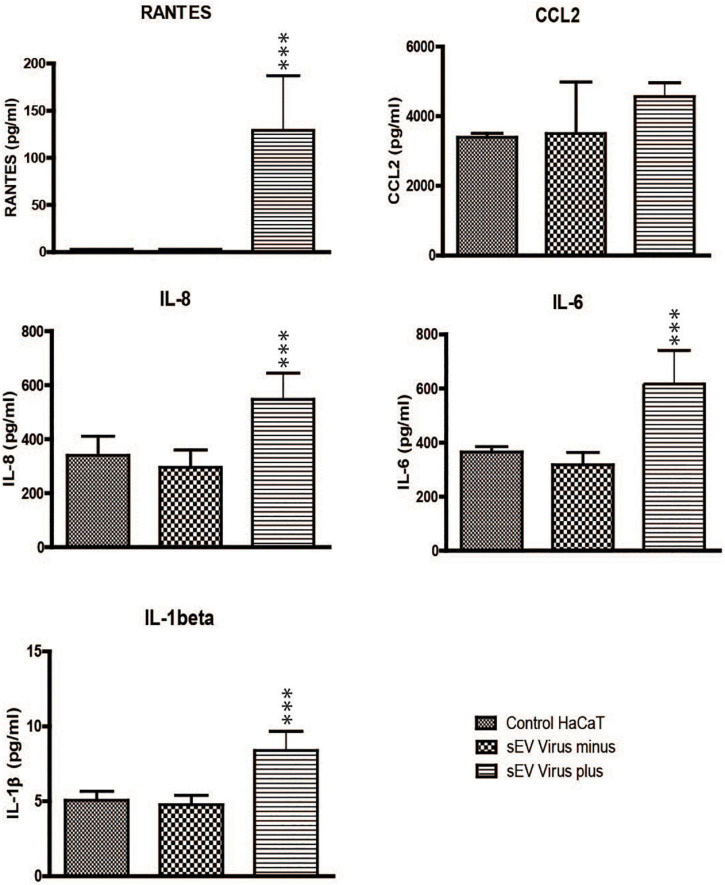
Small EVs from TV79-49c1^+^ stimulate a proinflammatory response in HaCaT cells. HaCaT cells were incubated with sEVs from TV79-49c1^–^ or Tv79-49c1^+^ for 24 h and subsequently levels of immune mediators were determined in a conditioned medium using ELISA. Medium without sEVs was used as a negative control. ***p-value < 0.0001.

*Trichomonas vaginalis* sEVs were shown to modulate host-parasite interactions (Twu et al., 2013; Govender et al., 2020). Thus, we were interested in whether and how the sEVs from TV79-49c1^+^ modulate the immune response of human cells in comparison with sEVs derived from TVV minus TV79-49c1^–^. To address this question, we co-incubated human keratinocytes (HaCaT cell line) with TVV plus and TVV minus sEVs for 24 h and quantified the production of five proinflammatory markers, including C-C motif chemokine ligand 2 (CCL2, syn. MCP-1), and RANTES (syn. CCL5) that are known to respond to dsRNA (Goodman et al., 2011; Sokol and Luster, 2015), proinflammatory cytokines IL-6 and IL-8 that were shown to be suppressed by sEVs from TVV positive *T. vaginalis* (Govender et al., 2020), and IL-1β, a proinflammatory factor during viral infection (Poeck et al., 2010). An advantage of HaCaT cells is that they are spontaneously immortal cells (Boukamp et al., 1988), without transformation with human papillomavirus oncogenes E6 and E7, which are often used for immortalization of various cell lines (Fichorova et al., 1997), and may interfere with signaling pathways involved in immunity and other cellular processes (Bienkowska-Haba et al., 2017). TV79-49c1^+^ EVs stimulated production of RANTES, while its production upon incubation with TV79-49c1^–^sEVs was close to the baseline as for control cells. TV79-49c1^+^ EVs also induced a significant increase in the production of IL-8, IL-6, and IL-1β in comparison to TV79-49c1^–^ EVs (Figure 8). These results indicate that sEVs derived from TVV-infected *T. vaginalis* stimulate a proinflammatory response in human HaCaT cells

## Discussion

Exosomes derived from virus-infected cells may contain various viral components with different functions together with altered protein and RNA cargo of host cell origin (Crenshaw et al., 2018; Saad et al., 2021). For example, the presence of viral RNA and proteins was reported in exosomes secreted by Rift Valley Fever Virus (RVFV) infected cells, although they do not serve for the virus transmission (Ahsan et al., 2016), while viruses such as hepatitis C virus (HCV), and porcine reproductive and respiratory syndrome virus (PRRSV) can infect naive cells *via* naked viral RNA within exosomes (Longatti et al., 2015; Wang et al., 2018). Other viruses include human polyomavirus 2, (Morris-Love et al., 2019), Dengue virus (Reyes-Ruiz et al., 2020), Rotavirus (Iša et al., 2020), and Norovirus (Todd and Tripp, 2020) exploit exosomes for transmission of the whole virions. Thus, we initially addressed the question of whether and if so, which viral components are present in sEVs derived from *T. vaginalis* that harbor TVV1, TVV2, and TVV3. Proteomic analysis of sEV cargo revealed the presence of peptides corresponding to TVV1, TVV2, and TVV3 capsid proteins and TVV1 RdRp. Deep RNA sequencing identified viral RNA fragments that covered a large part of TVV genomes including the 5′-UTR, and 3′-UTR, and complete ORF for RdRp and capsid protein were amplified from exosomes by reverse transcription PCR. This analysis encouraged us to investigate isolated sEVs by electron microscopy, which led to the visualization of viral particles within sEVs. Altogether, these experiments provided clear evidence that TVV particles are released in sEVs from infected *T. vaginalis* cells to the extracellular environment.

Trichomonasviruses and other dsRNA Totiviridae viruses are transmitted vertically during the protist division. How TVV-free *T. vaginalis* is infected remains unknown, and attempts to infect trichomonads with isolated TVV virions were unsuccessful (Wang and Wang, 1986). Our results raised another possibility for TVV transmission *via* sEVs as exosomes have been shown to incorporate into *T. vaginalis* membrane and transfer pathogenic factors between *T. vaginalis* strains (Twu et al., 2013). However, co-cultivation of TVV-free (acceptor) trichomonad clones with neither isolated TVV-plus sEVs, nor donor TVV-infected *T. vaginalis* cells led to the stable TVV infection. Although TVV genomic RNA was detectable in acceptor cells after selective removal of TVV-donor cells, it gradually declined within 6 weeks. This is reminiscent of recent experiments with related dsRNA LRV1 virus that infects *Leishmania guyanensis* (Atayde et al., 2019). Similar to TVVs, LRV1 particles were found in exosomes derived from infected leishmania and their genomic RNA was transferred among *Leishmania* species *via* exosomes. However, the transfer was only transient and LRV1 genome was cleared from the cells within two weeks (Atayde et al., 2019). An inability of exosomal TVV particles to infect trichomonads suggests that the particles may contain an incomplete or degraded genome. This is supported by the identification of multiple short TVV RNA fragments in sEVs, which is consistent with genomic RNA degradation. However, we were able to amplify also large parts of the genome over 2000 bp, thus the presence of a complete genome cannot be excluded.

Even though sEVs seem not to serve for TVV transmission, the release of TVV particles from *T. vaginalis via* sEVs allows their exposition to human host cells and may provoke immunomodulatory responses. Indeed, our experiments demonstrated that TVV-plus sEVs stimulate HaCaT cells’ production of RANTES, which is a key proinflammatory chemokine responding to viral infections (Fichorova et al., 2012). The proinflammatory response was further supported by the significant increase in the production of IL-8, IL-6, and IL-1β. An increase in IL6 and IL8 production was previously observed upon the interaction of human cervical epithelial cells with *T. vaginalis*-derived exosomes, however, the status of *T. vaginalis* viral infection in these experiments was not clarified (Twu et al., 2013). Sensing of TVV virus *via* Toll-like receptor 3 (TLR3), which triggers the proinflammatory response has been shown upon the interaction of several epithelial cell lines with TVV-infected *T. vaginalis* strains, isolated TVV virions, and polyinosinic:polycytidylic acid, a synthetic analog of double-stranded RNA (Fichorova et al., 2012). In contrast, sEVs derived from TVV- infected *T. vaginalis* were reported to have an immunosuppressive effect (Govender et al., 2020). The increase in production of proinflammatory cytokines (IL8, IL6, TNFa, nuclear factor kB, and RANTES) was observed only upon interaction with sEVs derived from the TVV-negative strain. Interestingly, TVVs was apparently not present in sEVs derived from TVV- positive strains in the study by Govender et al. (2020), as their proteomic analysis did not identify proteins of TVV origin in sEVs. These contradictory observations might be explained by differences in the biology of *T. vaginalis* strains and methods for the preparation of isogenic lines. In our study, we used a clonal population of *T. vaginalis* TV79-49 that was cryostabilized immediately after isolation (Flegr et al., 1987), and we derived a TVV-negative isogenic clone by a short treatment (96 h) with the inhibitor of RNA-dependent RNA polymerase (Narayanasamy et al., 2021). *T. vaginalis* 347V- (TVV-negative) strain used by Govender et al. (2020) was derived from 347V+ (TVV-positive) strain by *in vitro* cultivation for at least 6 months (Wang et al., 1987). However, such long-term *in vitro* cultivation has a strong impact on trichomonad biology (Stepkowski and Honigberg, 1972). In our study we observed a significant upregulation of proinflammatory cytokines in HaCaT cells upon interaction with sEVs that enclosed TVVs, however, we cannot exclude the possibility that naked TVV particles were rarely observed in sEVs preparation in electron microscopy contributed to this response. However, regardless of TVV topology, our experiments clearly demonstrated that sEVs mediated extracellular release of TVV from *T. vaginalis* to the environment where they are available for interactions with human cells.

In addition to the presence of viral components in sEVs, TVV infection has a major impact on the protein content and RNA cargo of sEVs. The proteome of sEVs included in the total of 1633 proteins, and of these, TVV-plus sEVs revealed 12 significantly enriched and 8 unique proteins including 4 proteins of TVV origin, while TVV-minus sEVs contained 43 enriched and 12 unique proteins. An interesting example is an enrichment of TvBspA (TVAG_268070) in TVV-plus sEVs. There are 911 TvBspA genes that share leucine-rich-repeats at the N-terminal region and of these, 190 TvBspAs including TVAG_268070 possess a single membrane-spanning domain close to the C-terminus (Noël et al., 2010). These proteins are typically present in eukaryotic and prokaryotic mucosal microbes and were implicated in parasite adherence. Indeed, investigation of individual TvBspAs revealed localization of TvBspA TVAG_073760 on the cell surface, and its transcription was stimulated upon *T. vaginalis* binding to extracellular matrix proteins (Noël et al., 2010). Expression of recombinant TvBspA TVAG_240680 increased *T. vaginalis* adherence, although it localized predominantly to the endoplasmic reticulum and Golgi apparatus (Handrich et al., 2019). We demonstrated that TVAG_268070 localized to MVB-like structures in *T. vaginalis* and shed sEVs. The observed increased amount of TvBspA in the proteome of TVV plus sEVs, and upregulation of the corresponding RNA suggest that the presence of TVV may enhance the binding properties of exosomes, and consequently delivery of the sEV cargo to the host cells. So far, three putative 4-α-glucanotransferases found in the proteome of *T. vaginalis* exosomes were implicated in the binding of exosomes to host cell carbohydrates (Rai and Johnson, 2019). However, the mode of their association with the exosomal membrane remains unclear as they do not contain TMDs. Moreover, these proteins were not detected in the proteome of sEVs in this study, however, all three 4-α-glucanotransferases have been found in *T. vaginalis* lysosomes (Zimmann et al., 2021). Altogether, the exact molecular mode of sEV binding to the host cells needs further studies, for which TVV-upregulated TVAG_268070 and other TvBspAs identified in sEVs represent attractive candidates for surface adhesines.

Previous proteomic analysis of sEVs from three TVV-plus (347V +, UR1 clone, and OC8), and three TVV-minus (347V-, OC7, and B7RC2) *T. vaginalis* strains led to the identification of three proteins that were enriched in TVV-plus, and four proteins in TVV-minus sEVs (Govender et al., 2020). However, none of these proteins was identified in our dataset as well as in the *T. vaginalis* proteome of exosomes by Twu et al. (2013). Interestingly, the exosomal proteome by Twu et al. included a total of 215 proteins, with only two proteins that overlapped with these reported in Govender et al. (2020). We were unable to compare a complete dataset of the later study with our proteome as unique protein identifications were provided only for 45 proteins (Govender et al., 2020), nevertheless, of these, only two proteins were common with our dataset, while our dataset shared 102 proteins with that of Twu et al. (2013). Many factors caused variability in proteomic analyses of sEVs content, particularly the separation and enrichment methods used for the isolation of EVs. In our experiments, we used methods based on Twu et al. (2013) with two differences: we incubated cells for only 2.5 h instead of 4 h in serum-free TYM medium to optimize trichomonad integrity, and we used isotonic linear OptiPrep gradient instead of sucrose gradient for the final step of sEVs separation. Govender et al. grew *T. vaginalis* in the complete TYM medium over a period of 24 h and sEVs were separated from conditioned TYM using gel filtration. Our method minimized the risk of dying cells, and contamination with secreted proteins, and allowed for efficient gradient separation. Although the size of sEVs proteome in this study (1633 proteins) is considerably higher than in Twu et al. (215), it is closer to the recent analysis of the proteome of exosomes (1079–1415 proteins) shed by three Leishmania species (Atayde et al., 2019).

Deep sequencing of small RNAs isolated from TVV-plus and TVV-minus sEVs revealed a significantly higher amount of tsRNA in TVV plus sEVs. The most abundant fragments were about 21 nt in length that belongs to internal fragments derived from tRNA-Val (GAC), and 27–29 nt that mapped predominantly to 5′fragments of tRNA-Gly (GCC), and tRNA-Glu (TCC), and less frequently we mapped tsRNA to 3′tRNAs. The distribution of the dominant type of tsRNAs in sEVs was different in comparison to tsRNAs found in *T. vaginalis* cells (Wang et al., 2021). While in sEVs, tsRNA-Gly, -Glu, and -Val comprise over 90% of all tsRNAs, these tsRNAs represented only 56% in *T. vaginalis* cells and the cellular tsRNA were more evenly distributed between the tRNA types, from which they were derived (Wang et al., 2021). This difference suggests that the packaging of tsRNA to *T. vaginalis* sEVs is a selective process as previously proposed for other cell types (Guduric-Fuchs et al., 2012; Abramowicz and Story, 2020). Importantly, the packing seems to be modulated by the presence of TVV as a significantly higher amount of tsRNA was detected in TVV-plus sEVs. Moreover, sEVs also contained fragments of viral genomic RNA. As previous studies demonstrated that *T. vaginalis* EVs deliver RNA cargo to human cells *via* a caveolae-dependent process (Rai and Johnson, 2019; Artuyants et al., 2020), changes in protein, as well as RNA cargo upon TVV infection, have the potential to modulate the host cell response as observed for other RNA viruses. For example, sEVs from cells infected with RVFV may induce apoptosis in uninfected cells (Ahsan et al., 2016). Infection with Newcastle disease virus (NDV) increases selective packaging and secretion of various mRNAs *via* exosomes, which results in the reduction of expression of corresponding proteins within the infected cells (Xu et al., 2021). Exosomes shed by Epstein-Barr virus (EBV) -infected cells contain viral miRNA that influences multiple signaling pathways in neighboring cells (Meckes et al., 2010). Whether RNA of trichomonad and TVV origin delivered into human cell contribute to pathogenesis and if so what is their molecular target remains to be established.

Interestingly, over 70% of annotated reads (over 25% of total reads) in TVV-plus and TVV-minus exosomes mapped to 18S, 28S, and 5S rRNAs, while only 0.4% of rRNA reads were found in a previous study of *T. vaginalis* EVs (Artuyants et al., 2020). However, there is great variability in the abundance of EV small rRNAs in various cell lines (Abramowicz and Story, 2020). The abundance of small rRNA in our dataset is close to that in EVs from *Leishmania* species (*Leishmania donovani*, and *Leishmania braziliensis*; Lambertz et al., 2015), in which the EV rRNA ranged from 14 to 30%. Dominant contribution of small rRNAs (56–76%) was observed in EVs shed by *Trypanosoma cruzi* (Bayer-Santos et al., 2014). The latter study also showed that the abundance of small rRNAs in EVs is similar to that in *T. cruzi* cell lysate (58%). In this respect, the contribution of sEV rRNA in our samples is close to 35% of rRNAs that contributed to a small RNA population in the lysate of three *T. vaginalis* strains (Wang et al., 2021). What is the function of small rRNA is currently unknown, some of them might be functional, but others are likely non-functional degradation products (Corrado et al., 2021).

In conclusion, we demonstrated that TVV particles are released from infected *T. vaginalis* cells to the extracellular environment *via* sEVs and induced proinflammatory responses in human HaCaT cells. Considering the high prevalence of TVV-harboring *T. vaginalis* clinical isolates, the release of TVV *via* sEVs might be critical for the development of pathologies related directly to trichomoniasis, as well as other pathologies related to modulation of the immune response such as the increased risk of preterm birth, susceptibility to HIV-1, and human papillomaviruses in *T. vaginalis*-infected patients. Comparison of sEVs derived from isogenic *T. vaginalis* clones that are TVV-free or possess three TVV species revealed that the presence of TVV has a significant impact on sEVs cargo, concerning both the proteome and transcriptome. Observed differences require further studies to elucidate their impact on sEVs properties such as adherence to target membranes, and to establish whether and how altered sEVs cargo, particularly viral RNA and tsRNA alter gene expression in recipient human cells.

## Data Availability Statement

The data presented in the study are deposited in the ProteomeXchange Consortium via the PRIDE partner repository (Perez-Riverol et al., 2019) under accession number PXD031790 and in the European Nucleotide Archive under accession number PRJEB50674.

## Author Contributions

JT, IH, and PR designed the experiments. JT, KH, and AZ, analyzed proteomic data. AZ, PT, S-CO, C-YT, H-WL, C-HC, analyzed deep sequencing data. VB performed RNA deep sequencing and data analysis. RN, PR, IH, TS, and JH, performed the experiments. JT and PR wrote the manuscript. All authors reviewed the manuscript.

## Conflict of Interest

The authors declare that the research was conducted in the absence of any commercial or financial relationships that could be construed as a potential conflict of interest.

## Publisher’s Note

All claims expressed in this article are solely those of the authors and do not necessarily represent those of their affiliated organizations, or those of the publisher, the editors and the reviewers. Any product that may be evaluated in this article, or claim that may be made by its manufacturer, is not guaranteed or endorsed by the publisher.

## Footnotes

1 http://pfam.xfam.org, 19,179 entries
