# Supplementary material for: Double-Stranded RNA Viruses Are Released From Trichomonas vaginalis Inside Small Extracellular Vesicles and Modulate the Exosomal Cargo
Source: Front Microbiol. 2022 May 4;13:893692. doi: 10.3389/fmicb.2022.893692 (PMC9114709; doi:10.3389/fmicb.2022.893692)

Supplementary data

Supplementary Tables

Table S1. List of primers.

Table S2. List of proteins identified in the sEV proteome from TV79-49c1^{+} (TVV plus) and TV79-49c1^{-} (TVV minus) and comparison with other *T. vaginalis* proteomes (exosomes, surface proteins, membrane vesicles, and lysosomes).

Table S3. Mapping of TVV peptides identified in sEVs from TV79-49c1^{+} (experiments were performed in triplicate (A, B, and C).

Table S4. Comparison of mRNA, rRNA, and tRNA sEV cargos between TV79-49c1^{-}

and TV79-49c1^{+}.

Table S5. Distribution of tsRNA fragments in TV79-49c1^{-} and TV79-49c1^{+}.

Supplementary figures

Figure S1. Melting curves corresponding to RT–PCR detection of TVV1-3 in EVs from TV79-49c1^{+} and TV79-49c1^{-} cells after incubation with EVs for 5 subcultures (p1-5). TV79-49c1^{-} cells served as a negative control.Trichomonads with microvesicles were incubated in TYM medium for 24 h to reach cell density 1.5x10^{6} per ml, then 1x10^{6} cells were transferred to 10 ml of the fresh medium, and 9x10^{6} cells were harvested and used for RNA isolation (p1). The same procedure was performed for consecutive four subcultures (p2-5). A melting curve indicating the presence of TVV1 was found for p1 sample with the curve maximum corresponding to positive samples Tv79-49+vBirA and TVV containing microvesicles. The shift of melting curve maximum in p2-p5 indicates non-specific amplification. No melting curve corresponds to the amplification of TVV2 and TVV3.

Figure S2. Melting curves corresponding to RT–PCR detection of TVV1-3 during cocultivation of TVV-positive and TVV-negative *T. vaginalis* cell lines (see Fig. 2). Samples for RT-PCR were taken after indicated numbers of subcultures (p5-p40).

Figure 3. Small EVs produced by TVV negative *T. vaginalis* clone TV79-49c1^{-}. (A) Small EVs from TV79-49c1^{-} were isolated in parallel with those from TVV positive *T. vaginalis* clone TV79-49c1^{+} using liner OptiPrep gradient (Fig. 1) and each fraction was analyzed by western blotting to detect the Tsp1 marker protein. (B) The size was determined using multi-angle dynamic light scattering in the fractions 17, 18, and 19.

Figure S4. Mapping of TVV peptides identified in the proteome of sEVs from TV79-49c1^{+} toTVV capsid protein and RdRp of TVV species and variants.

Figure S5. Mapping of RNA fragments isolated from TV79-49c1+ sEVs in three independent experiments (A-C).

Figure S6. Size distribution of the tsRNAs in TVV- and TVV+ sEVs that were grouped according to tRNA type and anticodon.

Figure S7. Coverage of tsRNAs from sEVs of TV79-49c1^{-} (TVV-) and TV79-49c1^{+} (TVV+) that were mapped to corresponding tRNAs.

Figure S8. Coverage of RNA fragments derived from 16S, 28S, and 5.8S rRNA in sEVs from TV79-49c1^{-} (TVV-) and TV79-49c1^{+} (TVV+). A. Examples of rRNA degradation for all rRNA types. B. Read length distribution of all reads assigned to 5.8S rRNAs. C. Representative examples of read length distribution of specific 5.8S rRNAs, which show similar pattern as the total 5.8S rRNA length distribution with a 28 and 42 nt peaks. D. Coverage of 5.8S rRNAs showed in C.

Figure S1. Melting curves corresponding to RT-PCR detection of TVV1-3 in microvesicles from TV79-49c1+ and TV79-49c1¯ cells after incubation with microvesicles for 5 subcultures (p1-5). TV79-49c1- cells served as a negative control.

TVV1 Microvesicles

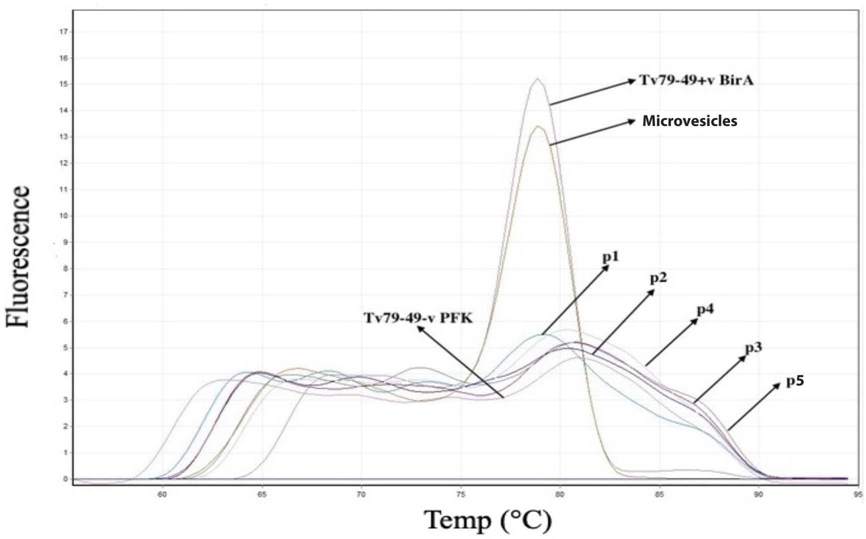

TVV2 Microvesicles

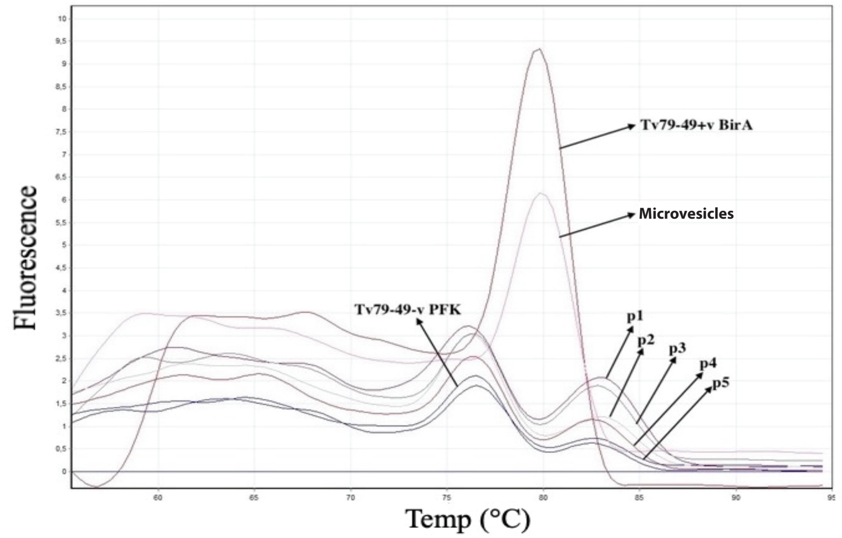

TVV3 Microvesicles

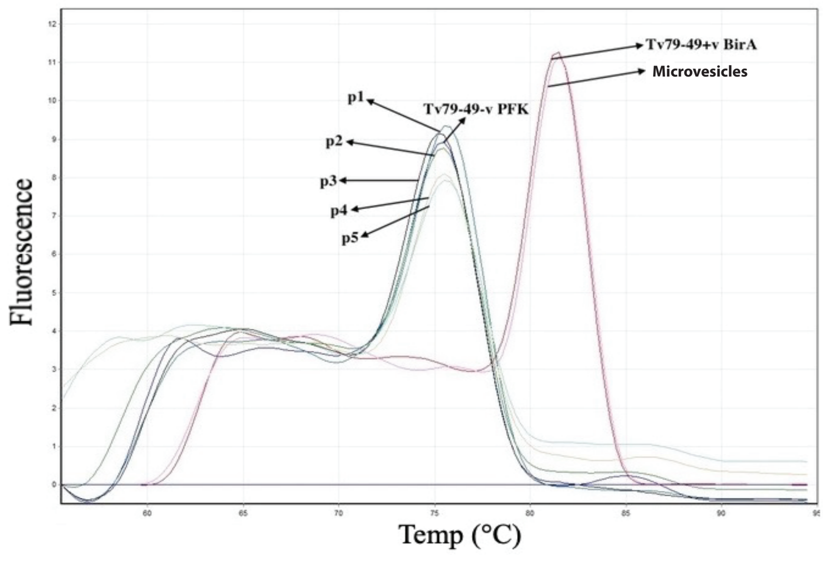

Figure S2. Melting curves corresponding to RT-PCR detection of TVV1-3 during cocultivation of TVV-positive (TV79-49c1^{+} PAC-BirA) and TVV-negative (TV79-49c1^{-} G418-PFK) *T. vaginalis* cell lines (see Fig. 2).

TVV1 cells cocultivation

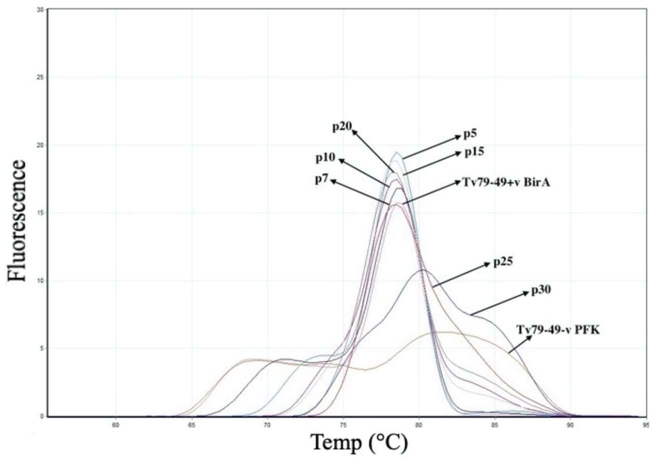

TVV2 cells cocultivation

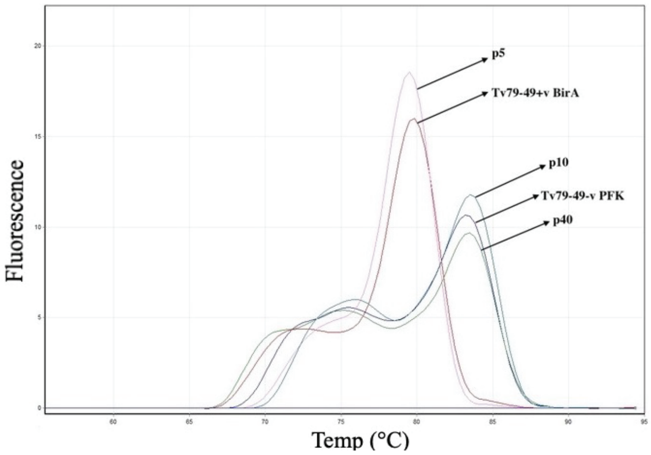

TVV3 cells cocultivation

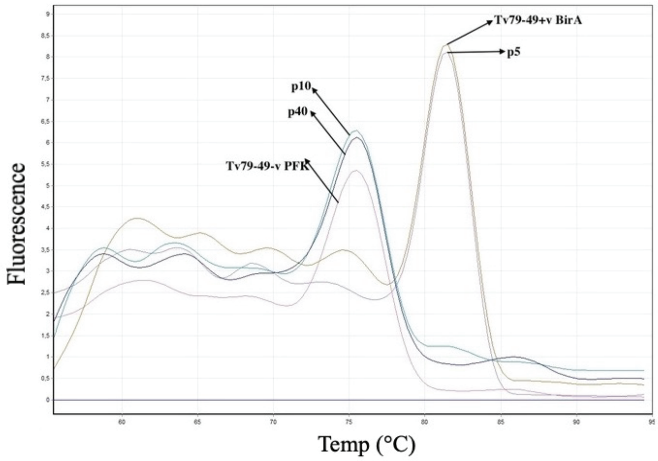

BirA

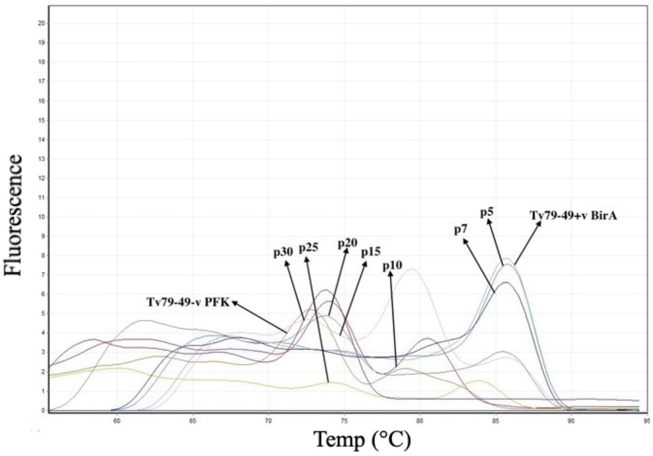

PAC

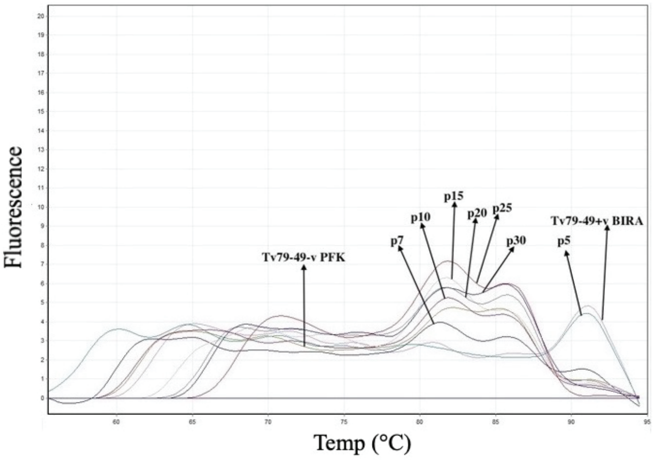

Figure 3. Small EVs produced by TVV negative *T. vaginalis* clone TV79-49c1$^-$.
(A) Small EVs from TV79-49c1$^-$ were isolated in parallel with those from TVV positive *T. vaginalis* clone TV79-49c1$^+$using liner OptiPrep gradient (Fig. 1) and each fraction was analyzed by western blotting to detect the Tsp1 marker protein. (B) The size was determined using multi-angle dynamic light scattering in the fractions 17, 18, and 19.

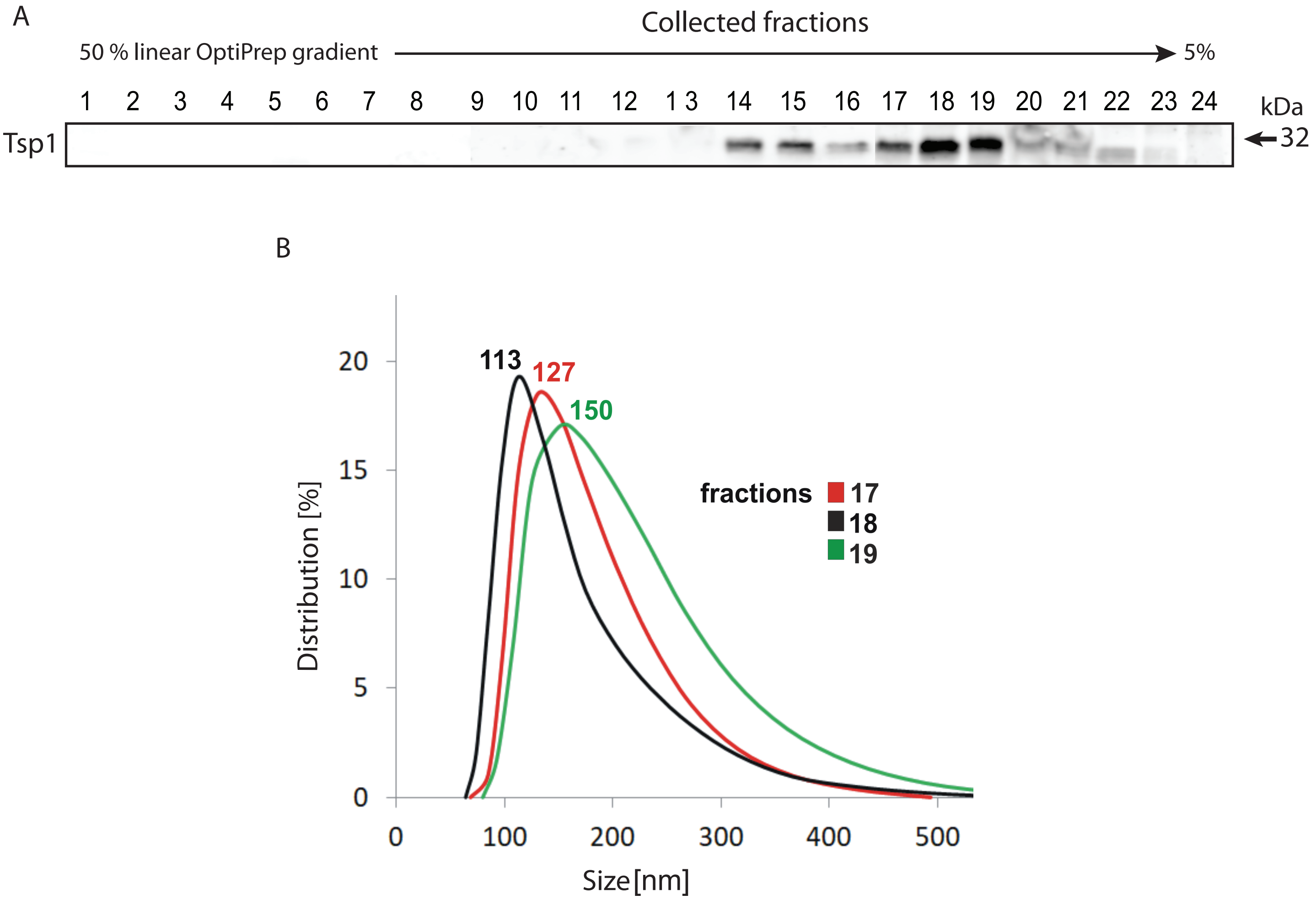

Figure S4. Mapping of TVV peptides identified in the proteome of sEVs from TV79-49c1+ to TVV capsid protein and RdRp of TVV species and variants.

```
                           10        20        30        40        50        60        70        80
                  ....|....|....|....|....|....|....|....|....|....|....|....|....|....|....|....|
AED99812.1_TVV1            MESSANGLSHDD-----NANKSQNVGPSTLPGSDKQG------------GENHENSFNSFSNDF-FFNFLR
ABC86750.1_TVV1            MEASARGLSHDD-----NANKSQNVGPSTLPRSDKQG------------GEKHEISFNSFSNDF-FFNFLR
AAC55468.1_TVV1            MEASANGLSHDD-----KANNSQNVGPSTLPGSDKQG------------GEKHENSFNSFSNDF-FFNFLR
AED99815.1_TVV1            MEASANGLSHDD-----NATRSQNVGPSTLPGSDKQG------------GEKHENSFNSFSYDF-FFNFLR
AED99805.1_TVV2        MASTLISSDNSATLGKVNEVINNTDTSPPDTSPGDHSNPRLTKILDEM-----SKKPCVNINEIRKMIRNFQPQI
AED99807.1_TVV2        MASTLISSDNSATSGTVGEVINNTDTSPPDTPPSDHSNPRLTKILDEM-----SKKPCVNINEIRKVIRNFQPQI
AED99801.1_TVV3   MSAPEPLNTEVRSPNGVS--------EATETQNLAVTQSSVSNEKTIDTQSDLQTLKKQLQPVTRSTDFETLYNYFYALN
AED99803.1_TVV3   MSAPEPLNTEVRSPDGVS--------EATETQNLAITQSRVSNEKITDTQSDLQTLKKQSQPVSRSTDFETLYNYFYGLD

                           90       100       110       120       130       140       150       160
                  ....|....|....|....|....|....|....|....|....|....|....|....|....|....|....|....|
AED99812.1_TVV1   TSTST-HISDSPGVSFVSKDGTP-YTSATIQSAVGRLTHNVVASAVQLNITANNTLEVDYGFGQDVSRA----TGTIT--
ABC86750.1_TVV1   MSTNT-HISDSPGVSFVGKDGTP-YSSATIPSAVSRLTHNVVAAAAQLNITSDNVLEVDYGFGQDVSRS----TGTIT--
AAC55468.1_TVV1   MSMNT-HISDSPGVSFIGKDGAP-YSSVTIQSAVGRLTHNVVASAVQLNVTADNVLEVDYGFGQDVSRA----TGTIP--
AED99815.1_TVV1   MSAHT-HISDSPGVSFVGKDGTP-YSTATIQSAVGRLTHNVIASAVQLNVTADNVLEVDYGFGQDVSRS----AGTIT--
AED99805.1_TVV2   IQPRN---GNRPANQPRTVDSFEWVVRIQSTVNTQLLGAT--------NTIPEQTLNLDISFTDD--------STTITPA
AED99807.1_TVV2   IQPRN---GNRPGAQPRTVNSFEWVVRIQSTVNTQLLGAT--------NTIPEQTLNLDISFTDD--------STTITPA
AED99801.1_TVV3   VSPSTDRIGNAITRNTPVNDTNE-VVSFPLTASVSHTFSN--------TPVPAHIQPLQISIADDCVNYELDESGTLCPA
AED99803.1_TVV3   VSPSTDRIGNAITRNTPVTDTNE-VVSFPLTASVSHTFSN--------SPVPAHIQPLQISIADDCINYELDESGTLCPA

                          170       180       190       200       210       220       230       240
                  ....|....|....|....|....|....|....|....|....|....|....|....|....|....|....|....|
AED99812.1_TVV1   -----IPIFDGEKYKEVARALSLVFSKKGMALDVTSQTVQDTLMNSDLTIATVAAGYYTALAARHELTKEASVAAHRIPF
ABC86750.1_TVV1   -----IPIFDGEKYKETARALAAIFSKKGSAVDVTSQTVQETLKNSDLTIATVAAGYYTALAARHELTKEVSLAQHTIPF
AAC55468.1_TVV1   -----IPIFDGEKYKETARALAMIFSKKGMSVDVTSQTVQETLKNSDLTIATVAAGYYTALAARHELTKAVSVAAHTIPF
AED99815.1_TVV1   -----IPIFDGEKYKETARALAAIFSKKGMAVDVTSQTVQETLKNSDLTIATVAAGYYTALAARHELTKEVSMAAHTIPF
AED99805.1_TVV2   SIPGSISMLDNSRHIPAIQSMIQNFKAR-----YLGNLQDTAQLNSPQYPQLLAYLFGQLIAIKDRLDLFR--PSNPLSF
AED99807.1_TVV2   SIPGSISMLDNSRHIPAIQSMIQNFKAR-----YLGNLQDTAQLNSPQYPQLLAYLFGQLIAIKDRLDLFR--PSNPLSF
AED99801.1_TVV3   --------LDSSVHVQRATSLASALKVK-----LTGEVMHSASVRPIQTPQLIAYLYGVLLAVQDRLNIHR---NQHTNL
AED99803.1_TVV3   --------LDSSVHVQRATSLASALKVK-----LTGEIMHSSSVRPIQTPQLIAYLYGVLLAVKDRINIHR---NQPTNL

                          250       260       270       280       290       300       310       320
                  ....|....|....|....|....|....|....|....|....|....|....|....|....|....|....|....|
AED99812.1_TVV1   VTALSDTFTAADNAQ------RSSHVISSCLRCPASNNAQRQVTVGTNMWTNVSVENLAVQGAAIPNPNDVSFFIPNKAL
ABC86750.1_TVV1   VTALSDTFTAARGAQ------RSSHVISSCLRCPASNNAQRDVAIGTNMWTNVFIESLSAQNMVVPNANDVSFFIPNKSL
AAC55468.1_TVV1   ATALSDTFTAAPNAQ------RSSHVISSCLRCPASGNIQHDIGIGSTIWTNVIVESLSPQNMAVPNPDDISFFIPNKAL
AED99815.1_TVV1   VTALSDTFSAAPDAQ------RSSHVISSCLRCPHSNNIQHDIGIGTDIWNNVSVESLSPQNMAVPNPNDVSFFIPNKAL
AED99805.1_TVV2   ADALFG-FTLAQHAHPRYDDHRHAKACTGPIVIPAATNA----DCGPCGFVQIN----ANQGLTLP-LGACLFVNPETVN
AED99807.1_TVV2   ADALFG-FTLAQNARPRYDDHIHAKACTGPIVIPAATNA----DCGPCGFVQIN----ANQGLTLP-LGACLFVNPDTVN
AED99801.1_TVV3   WRSLCA-AGRAAQAKPFFDEIPNNKFRTGALLAPPLPDA----GFGPFPAEGLN----QNSKLDFK-SKGYIFYKQRTYN
AED99803.1_TVV3   WRSLCA-AGRAAQAKPFFDEIPNNKFRPGALVAPPLPEA----GFGPFPAEGLN----QNSKLDFK-AKAYVFYKQRTYN

                          330       340       350       360       370       380       390       400
                  ....|....|....|....|....|....|....|....|....|....|....|....|....|....|....|....|
AED99812.1_TVV1   PSSWWCAIWLLNAFLH----SFVAQTRFHIFITPGETYNLAPFTDADIYEAIPV---LLAMSKSSRPVPESVESMLYAY-
ABC86750.1_TVV1   PPSWWCAIWLLNAFLH----SFVAPTRFHIFITPGETYHLAPFTDADVYEAIPI---LLAMSKSSRPVPESVESMLYAY-
AAC55468.1_TVV1   PSSWWCAIWLLNAFLH----SFVAPTRIHIFITQGETYHLAPFTDSDVYEAVRF---LLAMSKSSRPMPESVESMLYAY-
AED99815.1_TVV1   PSSWWCAIWLLNAFIH----SFVAPTRFHIFIAPGETYHLAPFTDADIYEAIPI---MLAMSKAARPVPESVESMLYAY-
AED99805.1_TVV2   DQSFQDFLWLIFATHHRMPNQMQNDWPFALNIVSTCAAPGRQAPQAGQFTEARVKLALDTGHRILLSMFNDDEEALRYYQ
AED99807.1_TVV2   DQSFQDFLWLIFATHHRMPNQMQNNWPFALNIVSTCAAPGRQVPQAGQLTDARFHAALDTGHRILLSMFNDDEETLRYYQ
AED99801.1_TVV3   PDDMNRAFWFIWAIYNRMPEDFQNSYPLNITFCTSELPVQSPMPAADGISAEQCDRALVLLDKVILEFFNNDRKLAYYYV
AED99803.1_TVV3   PDDMNRAFWFIWAIYNRMPNDFQNSYPLNITFCTSELPVQSPMPTADGISAEQCDKALLLLDKIVLEFFNNDRKLAYYYV

                          410       420       430       440       450       460       470       480
                  ....|....|....|....|....|....|....|....|....|....|....|....|....|....|....|....|
AED99812.1_TVV1   --GTQMVIQPHSLYTEGGIIRKMIFTVPHLPAHGYFVTNAEYSRYMN-IAVPNDPRTAKDYIIGVGTGLLQVILAYQAAF
ABC86750.1_TVV1   --GTQMIIQPHSLYTEGGLIRKMIFTVPHLPAHGYFVTNSEFSRYMN-IAVPNDPRSAKDYIIGAGTGLLQIVLAYQAAL
AAC55468.1_TVV1   --STQMIIQPHSLYTEGGLIRRMIFTVPHLPAHGYFVTNSEFSRYMN-IAVPDDPRSAKVFVIGAGTGLLQIVLAYQAAF
AED99815.1_TVV1   --GTQMIIQPHSLYTEGGLIRKMIFTVPHLPAHGYFVTNSEYSRYMN-IAVPNDPRSAKDFIIGAGTGLLQITLAYQAAF
AED99805.1_TVV2   RKGIETMFRPCCFYTEGGLLRKATRYVSMVPLSGLYYYNGASSYVVSPIHTDAHPG------ITAAVESFVDIMVLQAVF
AED99807.1_TVV2   RKGIETMFRPCCFYTEGGLLRKATRYVSMVPLSGLYYYNGASSYIVTPIHTDAHPG------ITAAIESFVDIMVLQAVF
AED99801.1_TVV3   FKGCQFVMRPCSCYQEGGLIRKASRNVALRAFTGIYYLAGFAEQYANMISCATHPG------IIGALFQYVDTMVLQAVF
AED99803.1_TVV3   FKGSQFVMRPCSCYQEGGLIRKASRNVALRAFTGIYYLAGFAEQYANMISCASHPG------IIGALFQYVDTMVLQAVF
```

```
                    490       500       510       520       530       540       550       560
               ....|....|....|....|....|....|....|....|....|....|....|....|....|....|....|....|

AED99812.1_TVV1  SCGGPIALHWHANDAISHGMDTVAAAYLEGRYFTIPMAINVATNIAQYTTGVRADPQYKHSLDRILPRIFGPSTDTVFNF
ABC86750.1_TVV1  SCAGPIALHWHGNDAISQGMDTIATTYLQGRYFTVPIAANVVNNVAQYTTLVRADPEYRHTLERILPRIFGPSIDTIYNF
AAC55468.1_TVV1  SCAGPIALHWHANDAISQGMDTVASTYLQGRYFTIPMAVNVATNVARYTTTVRADPQYKRTLDRILPRIFGPSTDTIFEF
AED99815.1_TVV1  SCAGPIALHWHANDAISQGMDTIAETYLQGRYFTVPMAVAVATNVAQYTTLVRADPQYRHTLDRILPRIFGPSTDTVFNF
AED99805.1_TVV2  SFSGPKVVAAKVDANQIDASSVFGPAVAEGDGFVYDPRRPAPPLSAFYTEFIHR-PAEQRIFQMAMSQIYGSHAPLIIAN
AED99807.1_TVV2  SFTGPKVVAARADANQVDASSVFGPAVAEGDGFVYDPRRPAPPLSAFYSEFIHR-PAEQRIFQMAMSQIYGSHAPLIIAN
AED99801.1_TVV3  SLSGPKLVRFAAPPEYQGRHACPFSFVADENYWGIAPGSDAEPVGMYYMDIIQR-KAEHDLFTETFMDIYGSTASIICAN
AED99803.1_TVV3  SLSGPKLVRFAAPPEYQGRHACPFSFVADENYWGIAPGSNAEPVGMYYTEIIQR-KTEHNLFTETFMDIYGSTASVICAN

                     570       580       590       600       610       620       630       640
               ....|....|....|....|....|....|....|....|....|....|....|....|....|....|....|....|
AED99812.1_TVV1  IESAITSSWVS-INATKRNGRAR--------KFRTAFINRFHDPEFAYMFGITGNGIERMEGKVTSNIAQEVEYLTNGGD
ABC86750.1_TVV1  IESAISSSWTS-IDARKRNGRAR--------KFRTAFINRFHDPEFAYMFGITGNGIERMEGKVTSNIAQEVDYLLNGGD
AAC55468.1_TVV1  IESAISSSWVS-IDAVRRSGRAR--------KFRTAFINRFHDPEFAYMFGITGNGIERMEGKVTSNIAQEVDYLMNGGD
AED99815.1_TVV1  IESAISSSWVS-IDARRRNGRTR--------KFRTAFINRFHDPEFAYMFGITGNGIERMEGKVTSTISQEVDYLLNGGD
AED99805.1_TVV2  VINSIHNCKTKIVNSKLRTAFVRRPPGAPPLKADTAIINRFHDPELAYALGILADGIAPLDGTHEYNILDELDYLFNGGD
AED99807.1_TVV2  VINSIHNCKTKIVNNKLRATFVRRPPGAPHLKADTAIINRFHDPELAYALGILADGIAPLDGTHEYNILDELDYLFNGGD
AED99801.1_TVV3  IETSLFTSGTNVINKRMQNDFARDTPKPGTLRHQHAIINRFHEPEYAYRLGILADGIIPLGGSFEVDILKEAERLITGED
AED99803.1_TVV3  IETSLFTSGTEVINQRMQNDFARDTPKPGTLRHQHAIINRFHEPEYAYRLGILADGIIPLSGSFEVDILKEAERLITGED

                     650       660       670       680       690       700       710       720
               ....|....|....|....|....|....|....|....|....|....|....|....|....|....|....|....|
AED99812.1_TVV1  LRNCPILRTLKAAEAEETVTFM------CTGKIGSIFAIDGTMRTFK-RYQTIDLAELGWTSHGKVMKPYAFRAPVIQGI
ABC86750.1_TVV1  LRNCPVLRTLKAAEREGTITFM------CKEKVGSLFAIDGTVRVFK-RYETIDLAQLGWTSHGKVMKPYAFRAPIMQGM
AAC55468.1_TVV1  LRNCPVLRTLKAAERDETITFM------CKEKVGSIFAIDGTVRVLK-QYQTIDLSQLGWTSHGKVMKPYAFRAPVIQGI
AED99815.1_TVV1  LRNCPVLRTLKAAERDETITFM------CKEKAGTLIAMDGTVRFFK-RFETIDLTQLGWTSHGKVMKPYAFRAPLINGI
AED99805.1_TVV2  IRNCFGLNALNTRGLGQIVHVRPKREPGKRPRRGYYTTLDGQVHSVTQDAPLDEIYH--WRDHGNLTRPYSCHILDSEGL
AED99807.1_TVV2  IRNCFGLNALNTRGLGQIVHVRPKRDPGKKPRRGFYTTLDGQVHPITHDAPLDEIYQ--WRDHGNLTRPYSCHILDSEGL
AED99801.1_TVV3  IRNLPGLRCLCSRGLDAILGIRPVQQ--KRKKMSYFRTLDGTFHEVTIRSDTHDLQV--WNDHGYLARPYACHIVDSDGI
AED99803.1_TVV3  IRNLPGLRCLCSRGLDAILGLRPIQQ--KRKKMCYFRTLDGNFHEVTIRSETRDLQV--WRDHGYLARPYACHIVDSDGI

                     730       740       750       760       770       780       790       800
               ....|....|....|....|....|....|....|....|....|....|....|....|....|....|....|....|
AED99812.1_TVV1  TVCKTAYTSTAIDIVTTVFGPL---RLRVGTLLSKAVRCGPIIPSVKHHFNIRRIITVKRNGNEYVFIPGYGWVLQDDYL
ABC86750.1_TVV1  TICNTAYTSTDIDVVTTVFGPL---RYHVGALFE-------------------------------------------
AAC55468.1_TVV1  TICNTAYTTTAIDIVTTVFGPL---RQRVGTLFE-------------------------------------------
AED99815.1_TVV1  TICNTAYTTTAIDIVTTVFGPL---RQRVGSLFE-------------------------------------------
AED99805.1_TVV2  QFADVSNGRTRGKILVVVNTPLKTSAAYQGPSFAPKPGSAMWNE-----------------------------------
AED99807.1_TVV2  EFADVSNGRSRGKLLVVVTTPLKTSAAYQGPSFAPKPGSAMWNE-----------------------------------
AED99801.1_TVV3  EFYDKSNGLYKGRVNVLISGFAIPGRAYRGPRLPVATEAAQV-------------------------------------
AED99803.1_TVV3  EFYDKSNGLYKGRVNVLISGFAIPGRAYQGPRLQVATEAAQI-------------------------------------

                     810       820       830       840       850       860       870       880
               ....|....|....|....|....|....|....|....|....|....|....|....|....|....|....|....|
AED99812.1_TVV1  VNSVKMTGEDQLPPNQLPYGDDLLLIYSEILLYNYISLFPKFRYKNPDLLNQETELQLFPLKTDSAARNKANFYARSLWN

                     890       900       910       920       930       940       950       960
               ....|....|....|....|....|....|....|....|....|....|....|....|....|....|....|....|
AED99812.1_TVV1  EAKTDKTAFKPGTYNDTVAGLLMWQQCALMWSLPRSVINRTISGVCDALTERTSLALLKRISDWLKQLGLACSPIHRLFI
                     970       980       990      1000      1010      1020      1030      1040
               ....|....|....|....|....|....|....|....|....|....|....|....|....|....|....|....|
AED99812.1_TVV1  ELPTLLGRGAIPGDSVKDMKHRLKFDPSITVDVPRDQLHDLIYRLLSRNLHITNVESFDHHLEERLLWSKSGSHYYPDEE
                    1050      1060      1070      1080      1090      1100      1110      1120
               ....|....|....|....|....|....|....|....|....|....|....|....|....|....|....|....|
AED99812.1_TVV1  VNRLLPNQPTRKEFLDVVTVDYIKECKPQVFIRQSRKLEHGKERFIYNCDTVSYVYFDFILKLFEAGWQDSEAILSPGDY
                    1130      1140      1150      1160      1170      1180      1190      1200
               ....|....|....|....|....|....|....|....|....|....|....|....|....|....|....|....|
AED99812.1_TVV1  TGERLHARISSYKYKAMLDYTDFNSQHTIRSMRLIFETMKELLPPETTFALDWCIASFDNMYTSDGHKWVSTLPSGHRAT
                    1210      1220      1230      1240      1250      1260      1270      1280
               ....|....|....|....|....|....|....|....|....|....|....|....|....|....|....|....|
AED99812.1_TVV1  TFINTVLNWCYTQMVGLKFNSFMCAGDDVILLSQEPISLVPILTSHFKFNPSKQSTGTRGEFLRKHYTSEGVFAYPARAI
                    1290      1300      1310      1320      1330      1340      1350      1360
               ....|....|....|....|....|....|....|....|....|....|....|....|....|....|....|....|
AED99812.1_TVV1  ASLVSGNWLSQSLRENTPILVPIQNGIDRLRSRAGLLGVPWILGLSELTEREAVPRDVSMALLNSHAAGPGLITRNYSSF
                    1370      1380      1390      1400      1410      1420      1430      1440
               ....|....|....|....|....|....|....|....|....|....|....|....|....|....|....|....|
AED99812.1_TVV1  TVTPKPPTLTSTLEYTATRYGVQDLSKHVPWEQLTLEERNKLGKQIKKMSHRHCSQAKITYTCVHEVYKPSGLPKVLSGA
                    1450      1460      1470      1480      1490      1500
               ....|....|....|....|....|....|....|....|....|....|....|....|
AED99812.1_TVV1  SQPSLSMVWWQAMLKEAMQDNSTKKIDAQMFASSACTDRVSGDAFLQASAKAAGVLITSLIQSSS
```

Complete proteome was mapped against 92 protein sequences of TVV1-4 that led to identification of unique peptides in four variant TVV1 sequences, two TVV2 sequences and 2 TVV3 sequences. Amino acid residues

in red indicate peptides that mapped to TVV capsid protein and RdRp. Residues in blue indicate unique sites.

Figure S5. Mapping of RNA fragments isolated from TV79-49c1+ sEVs in three independent experiments (A-C)

Mapping of RNA fragments isolated from sEVs from TV79-49c1+ to TVV1 genome, Experiment A
Total read count: 783
Length Coverage: 2131/4666
Average Depth:7.35

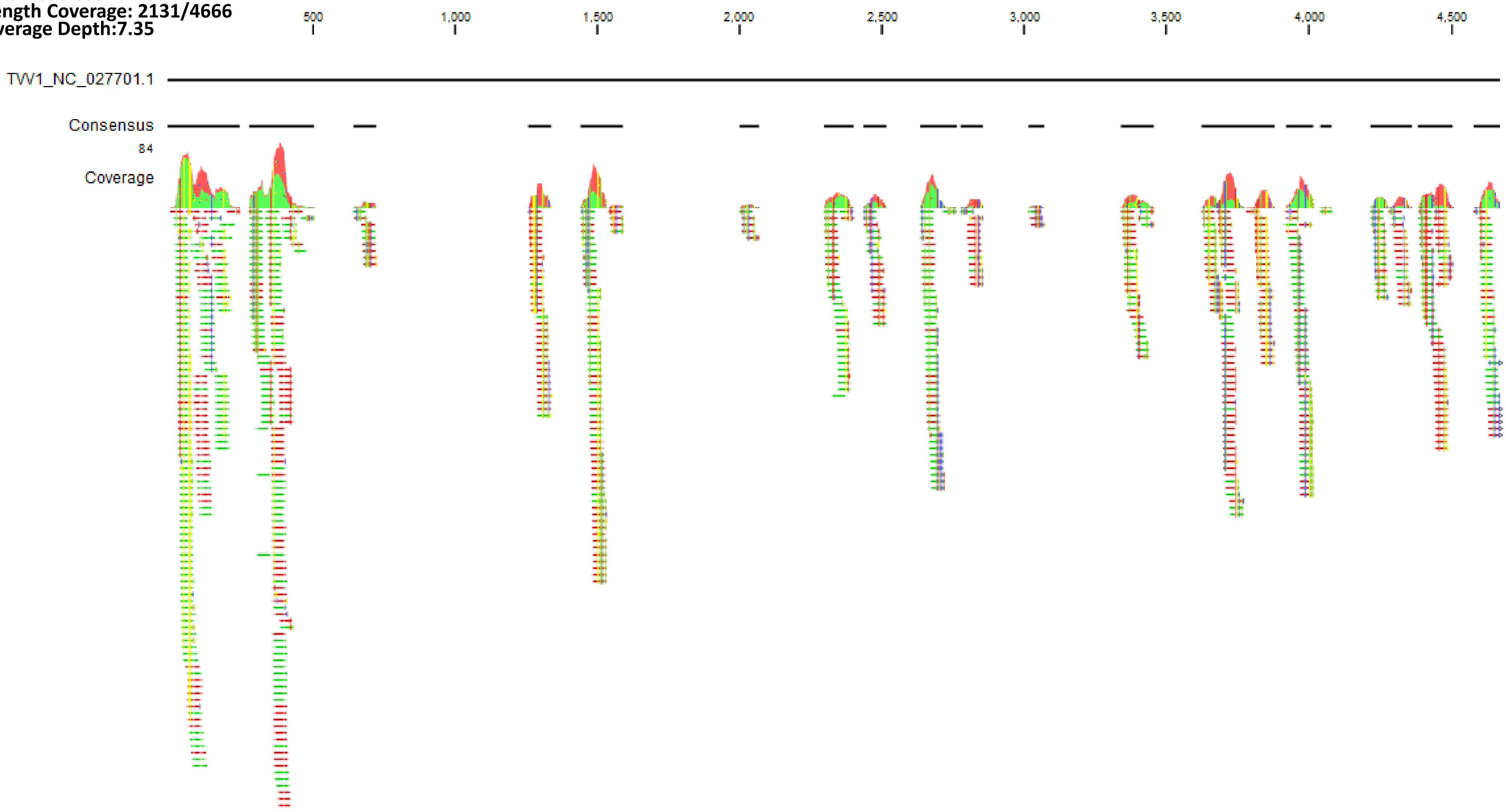

Mapping of RNA fragments isolated from sEVs from TV79-49c1+ to TVV2 genome, Experiment A
Total read count: 248
Length Coverage: 2380/4674
Average Depth: 2.31

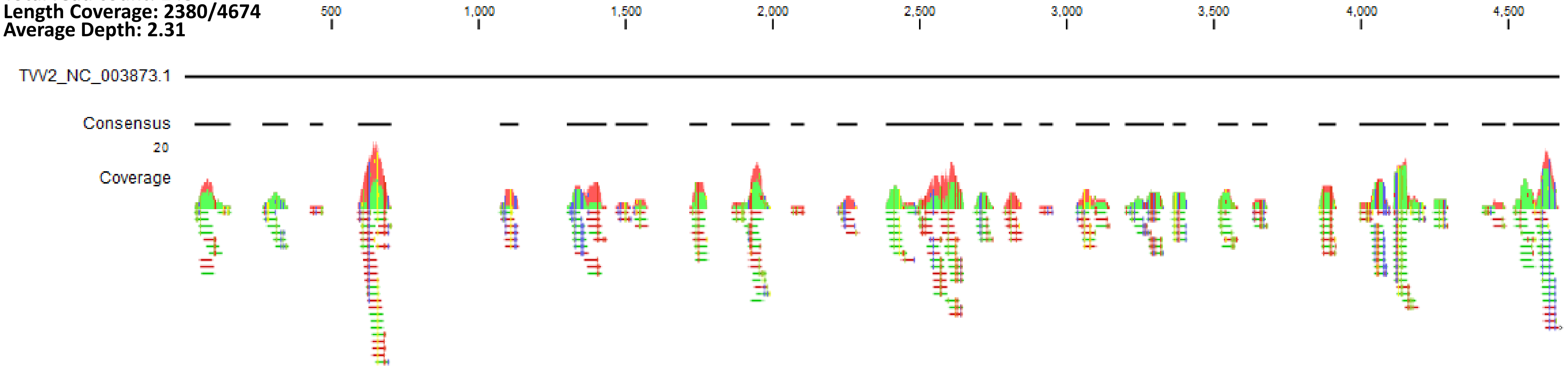

Mapping of RNA fragments isolated from sEVs from TV79-49c1+ to TVV3 genome, Experiment A
Total read count: 781
Length Coverage: 2729/4844
Average Depth: 7.03

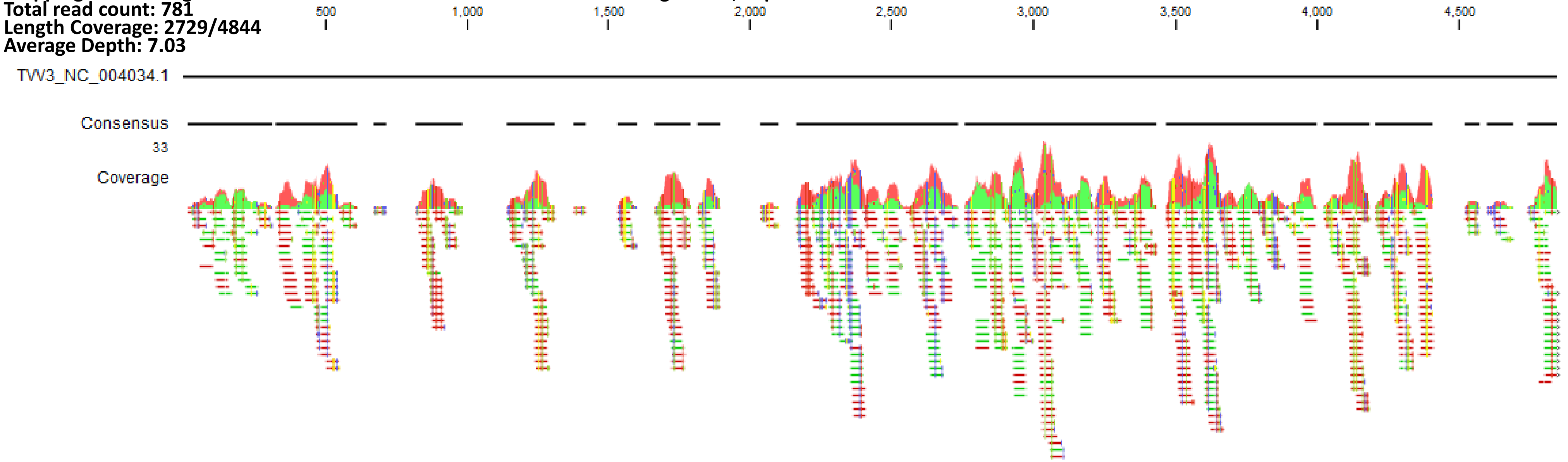

Mapping of RNA fragments isolated from sEVs from TV79-49c1+ to TVV1 genome, Experiment B
Total read count: 2880
Length Coverage: 2501/4666
Average Depth: 27.18

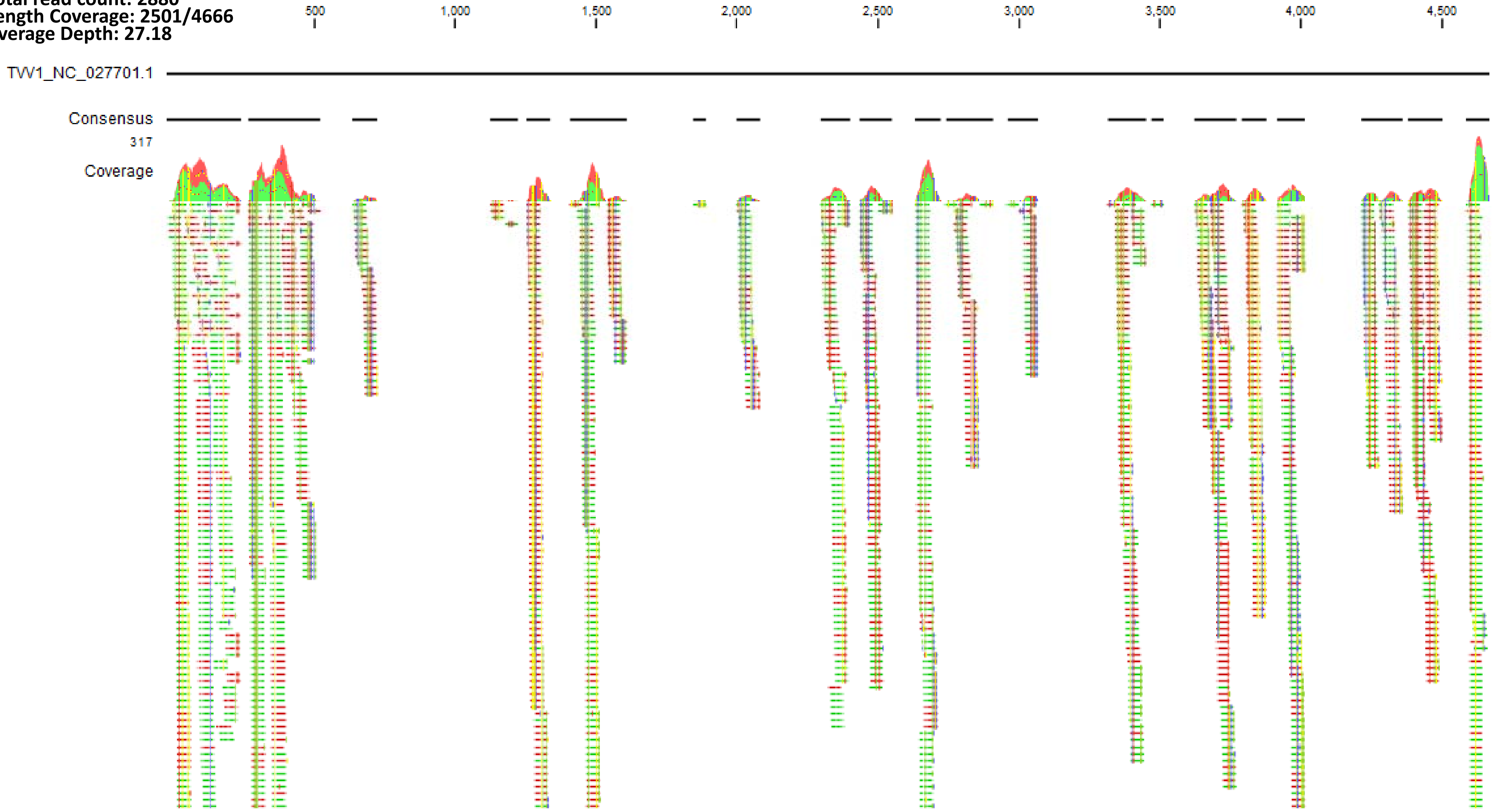

Mapping of RNA fragments isolated from sEVs from TV79-49c1+ to TVV2 genome, Experiment B
Total read count: 1480
Length Coverage: 2988/4674
Average Depth: 14.07

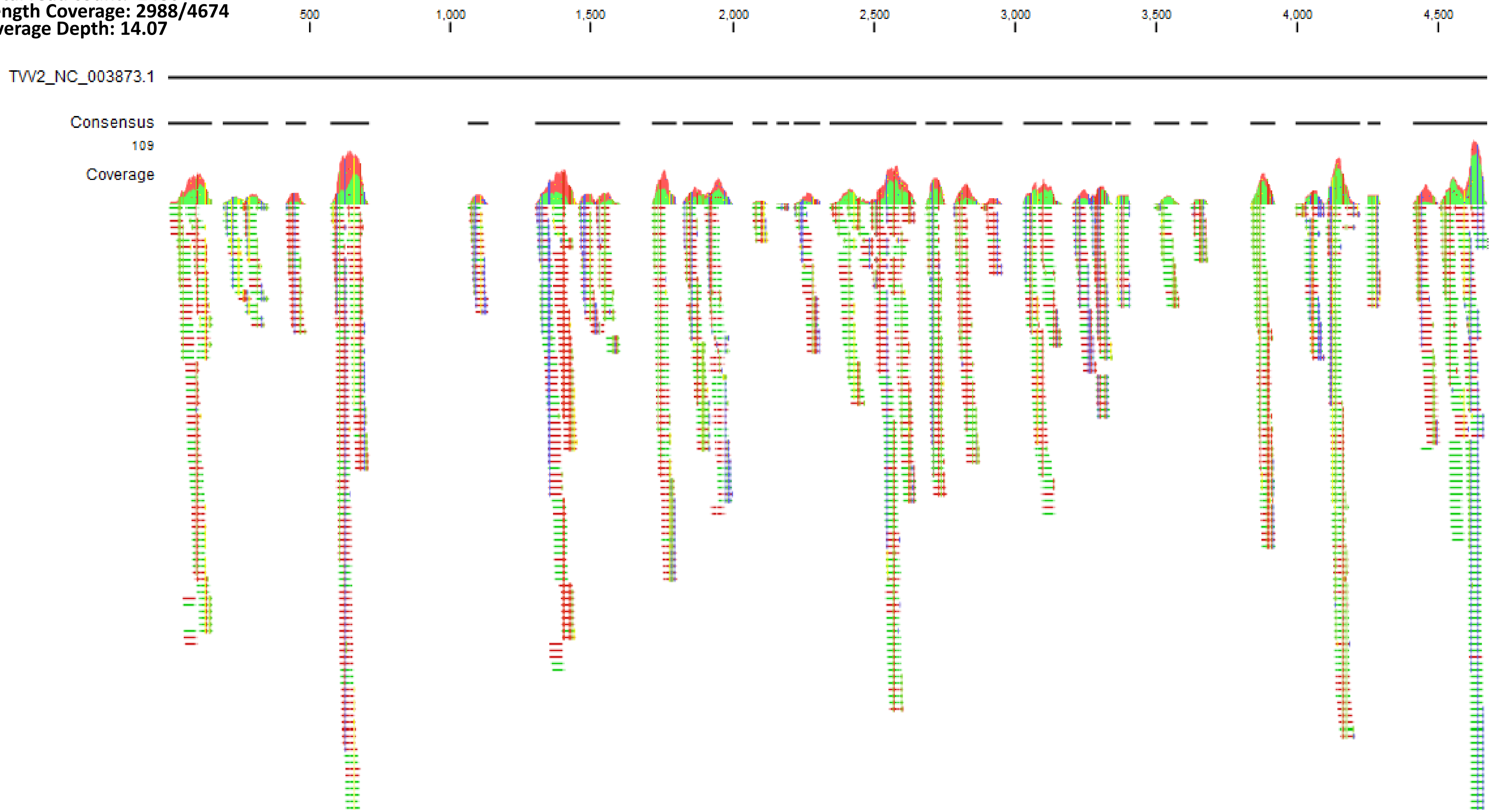

Mapping of RNA fragments isolated from sEVs from TV79-49c1+ to TVV3 genome, Experiment B
Total read count: 6029
Length Coverage: 4176/4844
Average Depth: 55.33

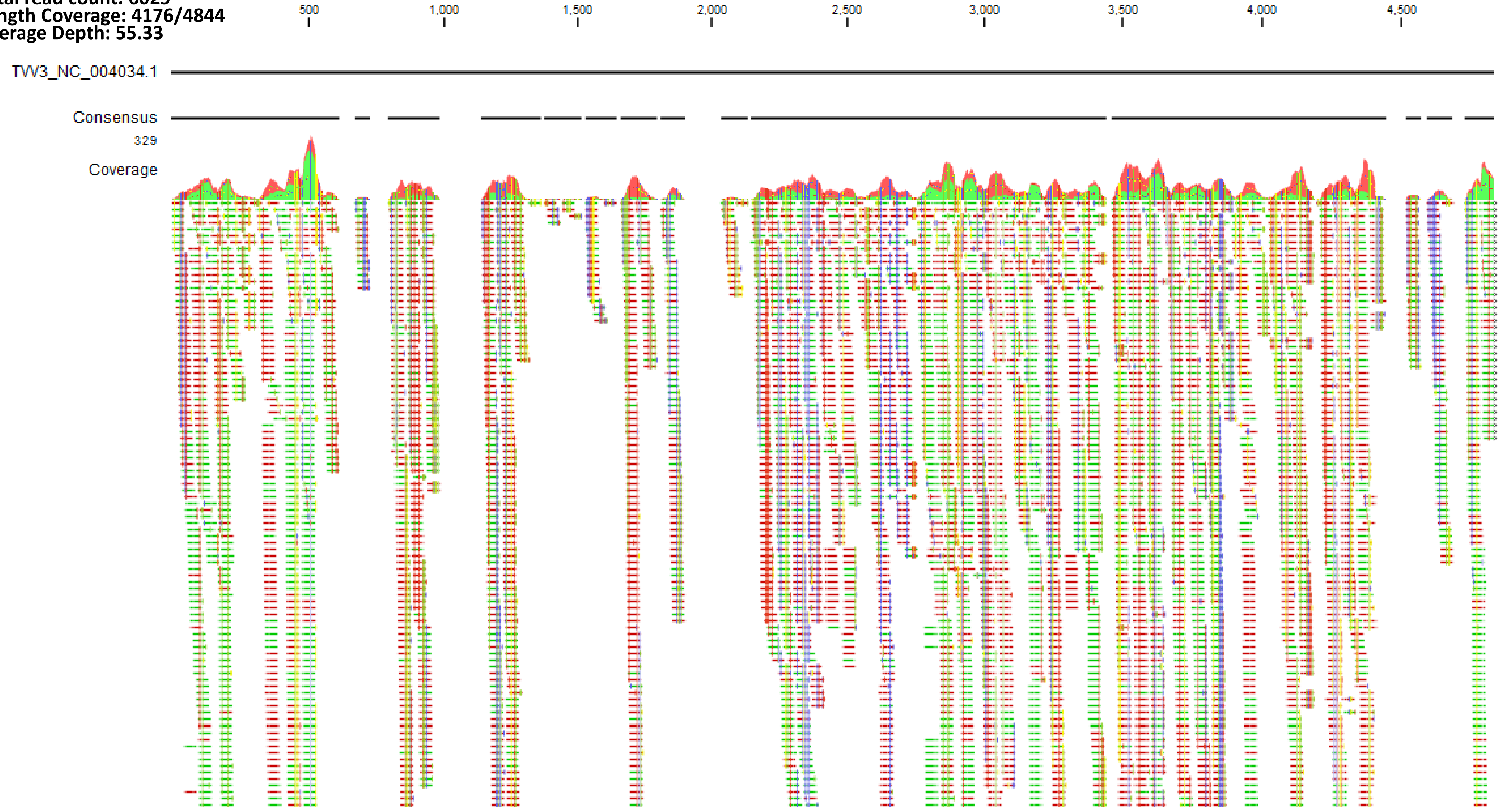

Mapping of RNA fragments isolated from sEVs from TV79-49c1+ to TVV1 genome, Experiment C
Total read count: 1202
Length Coverage: 2214/4666
Average Depth: 11.42

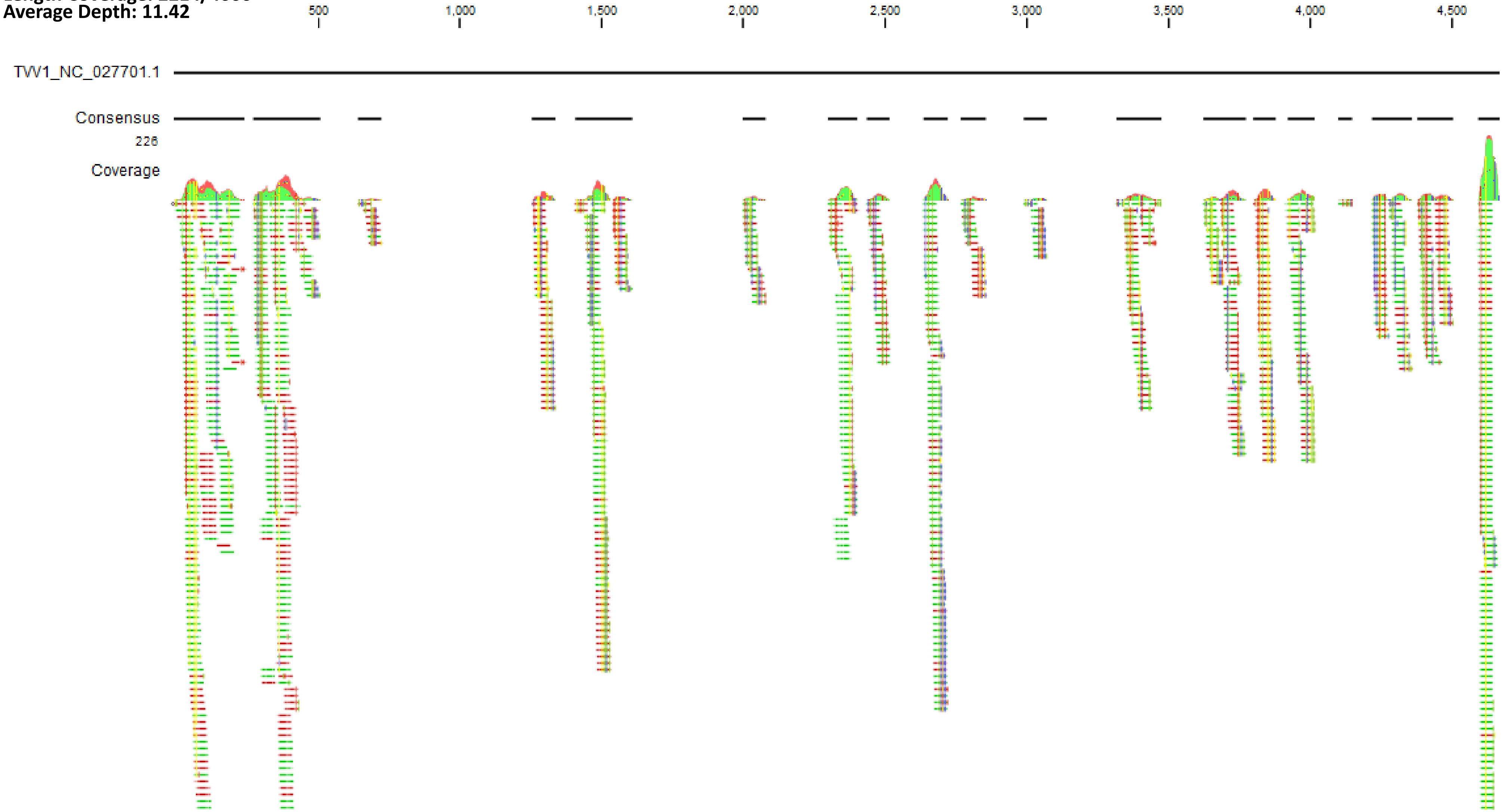

Mapping of RNA fragments isolated from sEVs from TV79-49c1+ to TVV2 genome, Experiment C
Total read count: 1059
Length Coverage: 2881/4674
Average Depth: 10.14

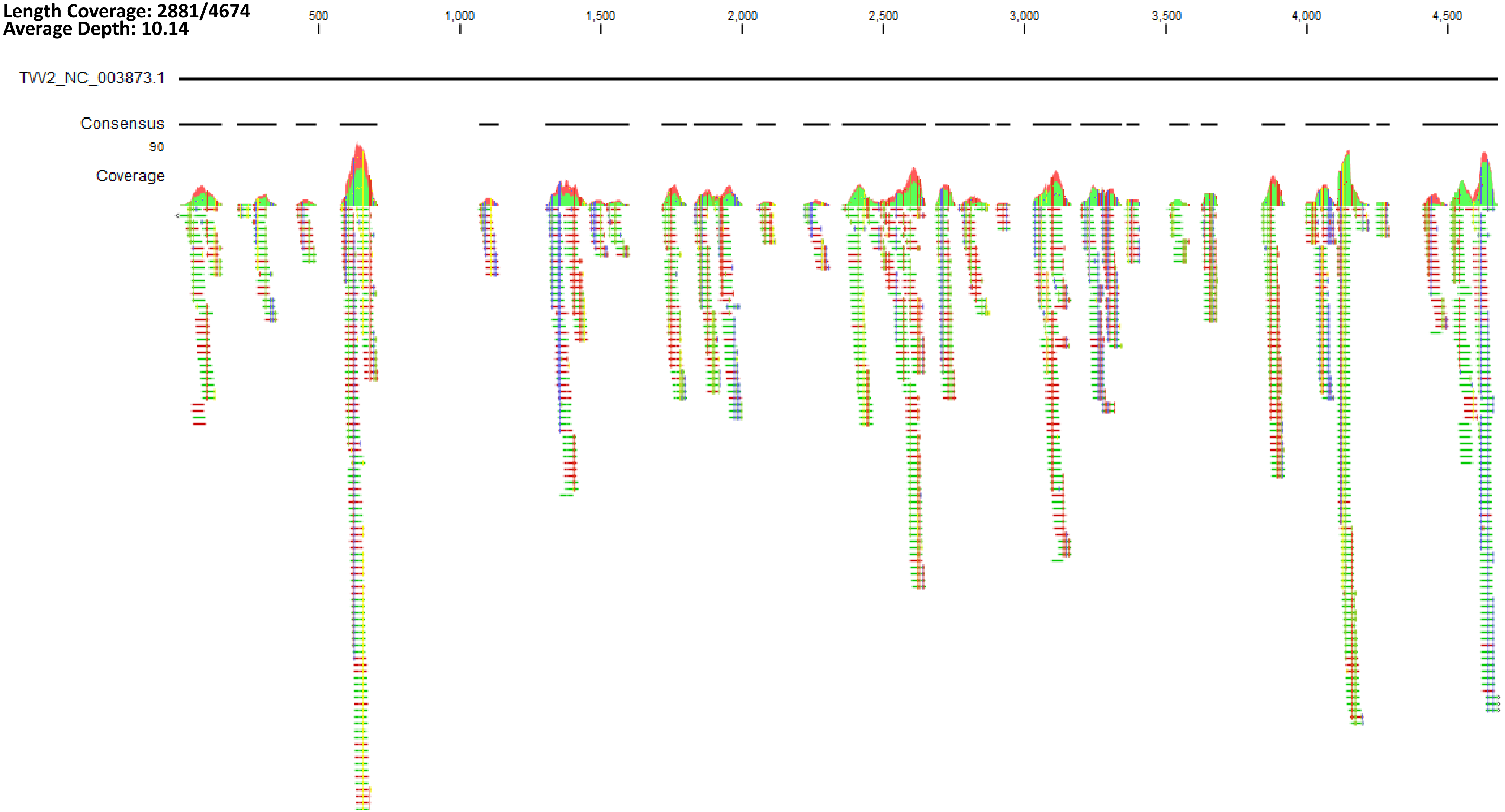

Mapping of RNA fragments isolated from sEVs from TV79-49c1+ to TVV3 genome, Experiment C
Total read count: 3094
Length Coverage: 3975/4844
Average Depth: 28.48

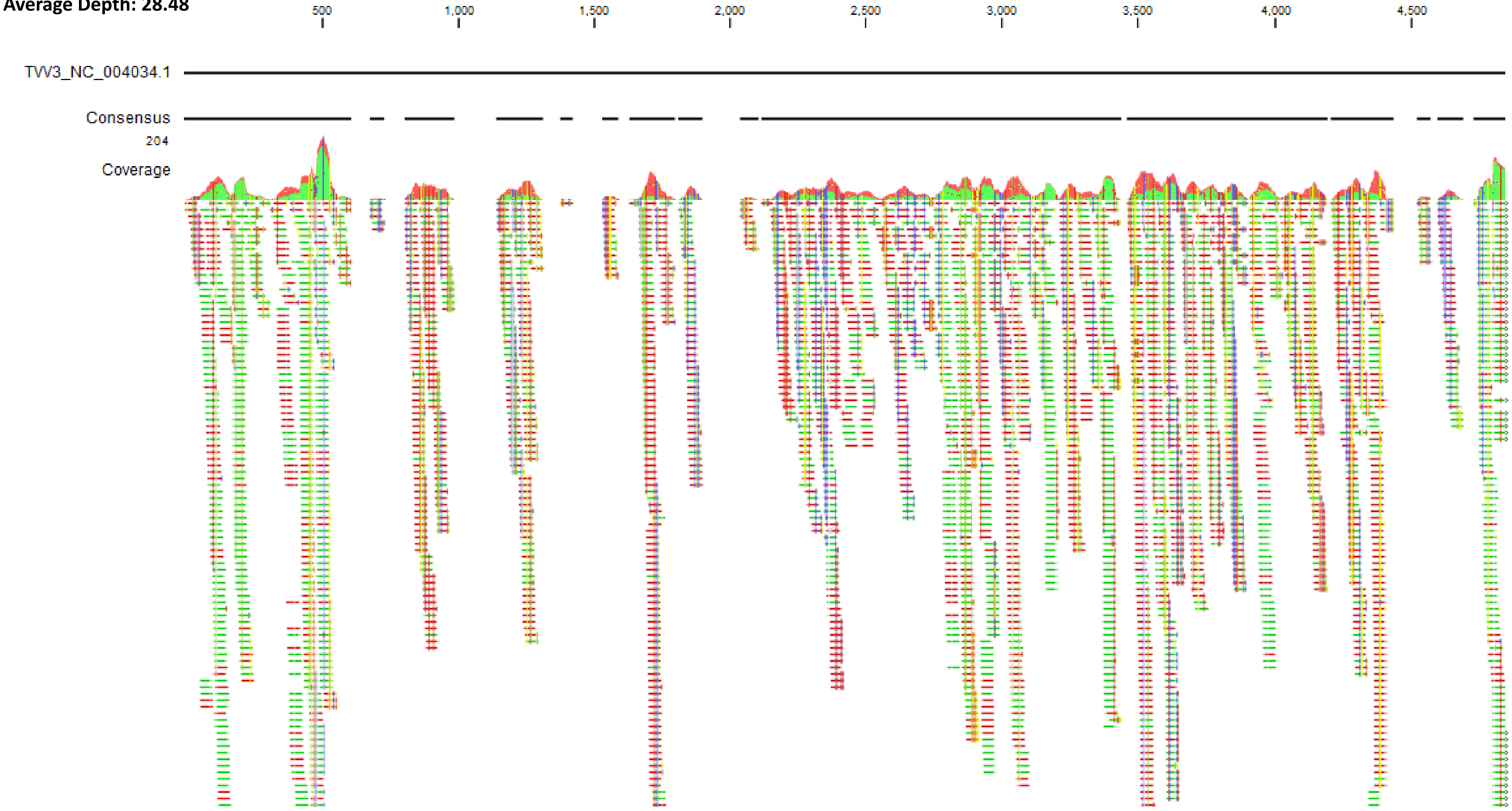

Figure S6. Size distribution of the tsRNAs in TVV- and TVV+ sEVs that were grouped according to tRNA type and anticodon.

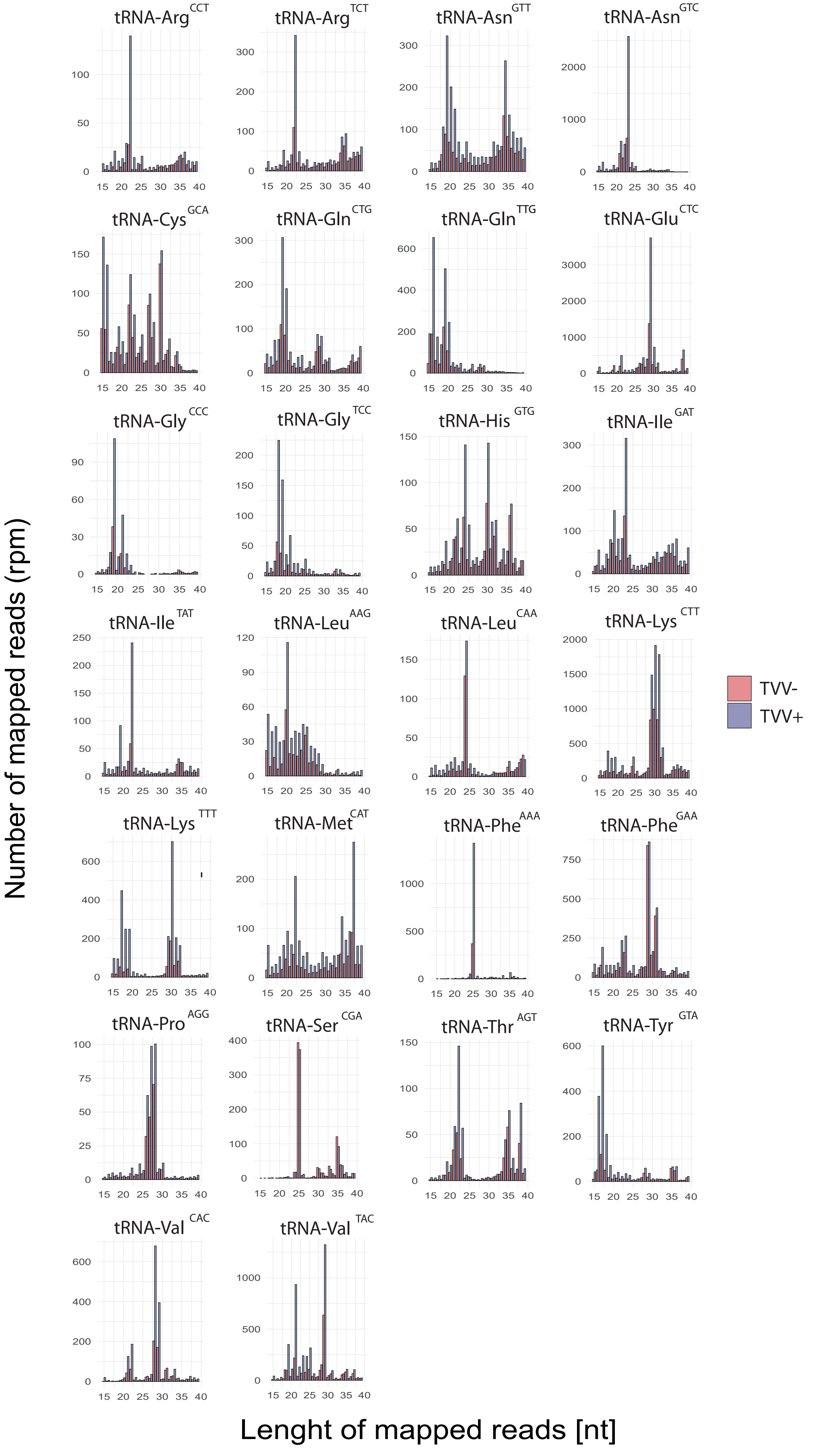

Figure S7. Coverage of tsRNAs from sEVs of TV79-49c1- (TVV-) and TV79-49c1+ (TVV+) that were mapped to corresponding tRNAs.

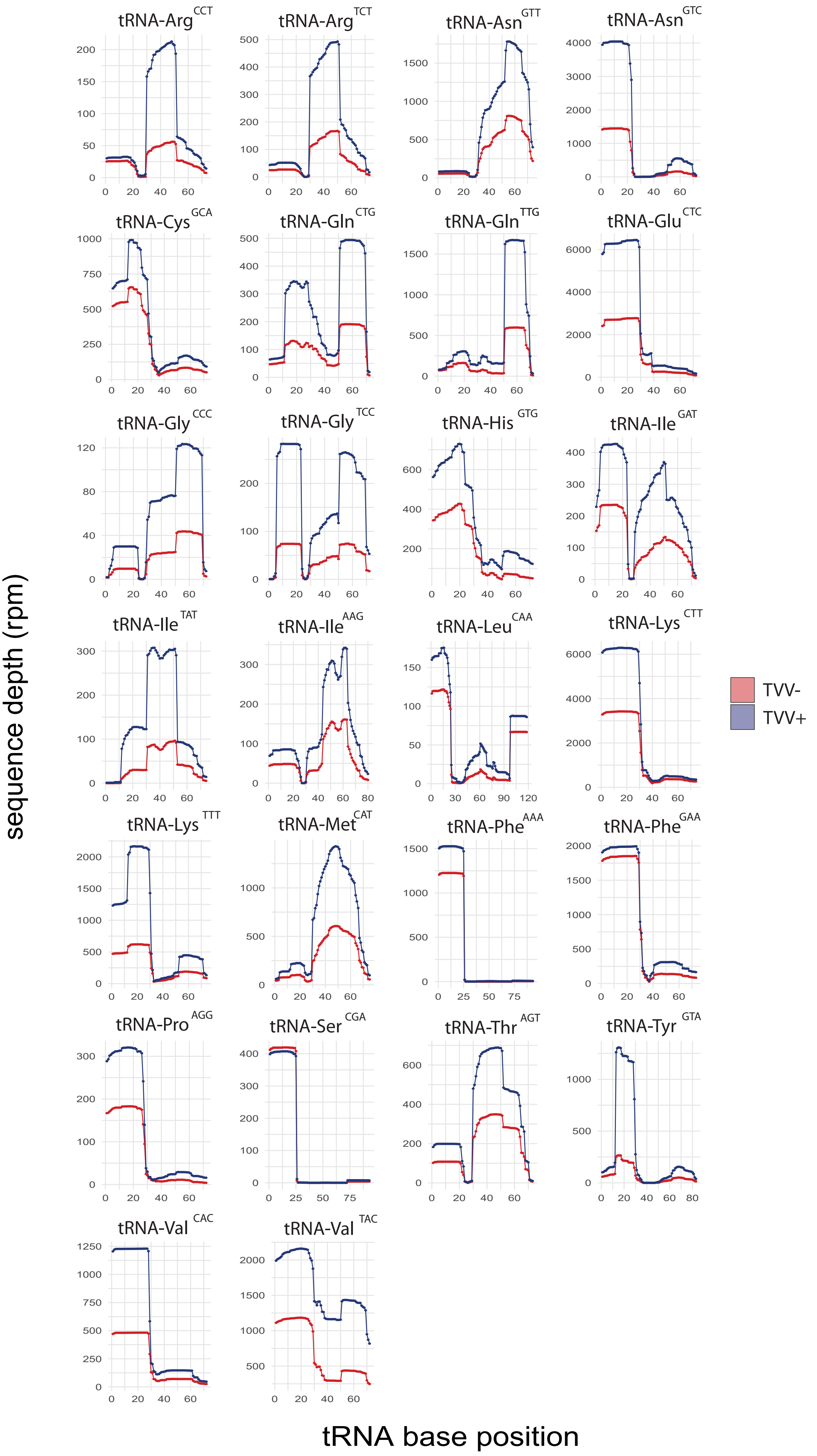

Figure S8. Coverage of RNA fragments derived from 16S, 28S, and 5.8S rRNA in sEVs form TV79-49c1- (TVV-) and TV79-49c1+ (TVV+).

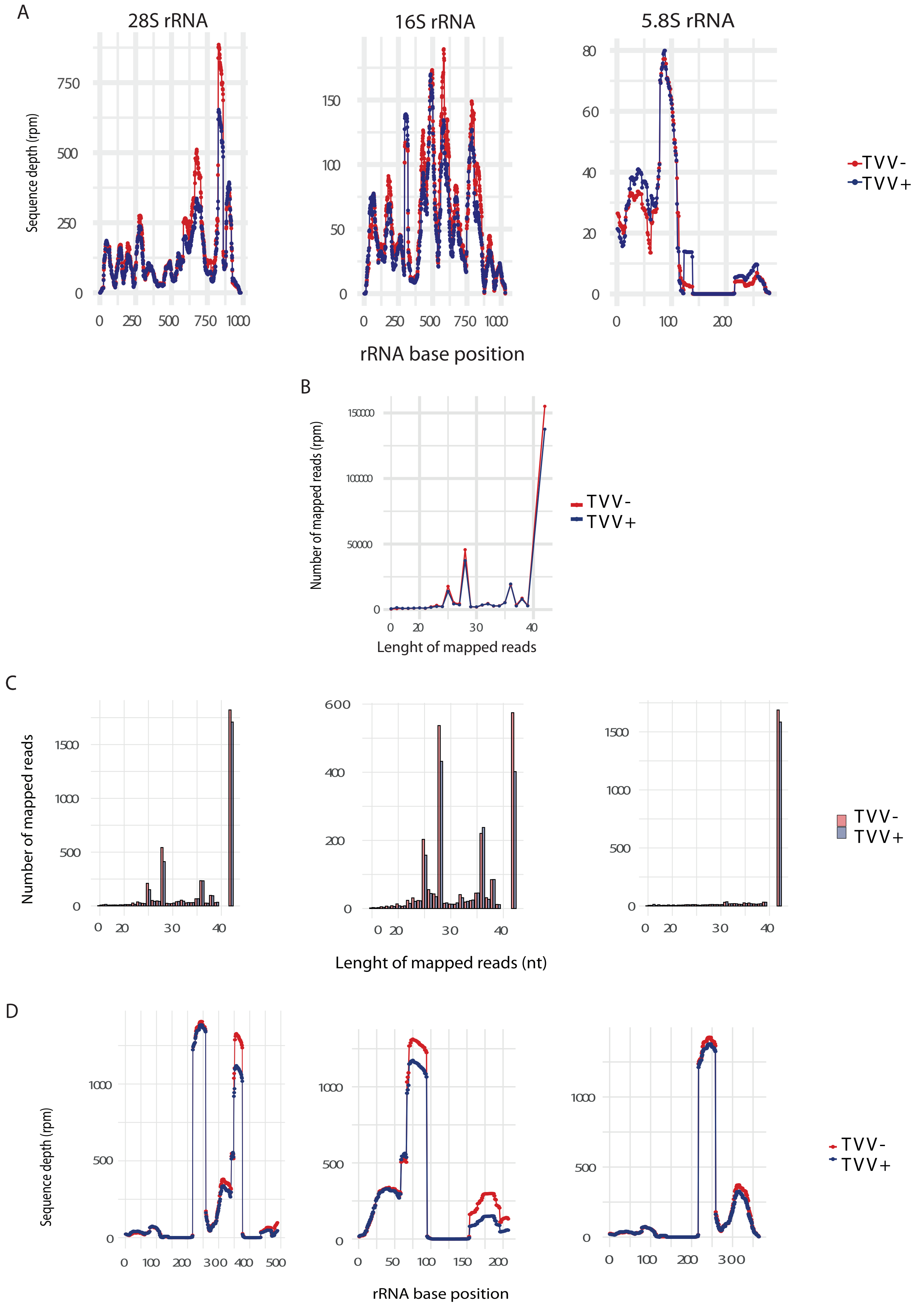

Supplement: Supplementary file 1 [file Data_Sheet_1.PDF]
